# Climate change leads to accelerated transformation of high‐elevation vegetation in the central Alps

**DOI:** 10.1111/nph.15290

**Published:** 2018-06-25

**Authors:** Andrea Lamprecht, Philipp Robert Semenchuk, Klaus Steinbauer, Manuela Winkler, Harald Pauli

**Affiliations:** ^1^ GLORIA Coordination Center for Global Change and Sustainability University of Natural Resources and Life Sciences Vienna & Institute for Interdisciplinary Mountain Research Austrian Academy of Sciences Vienna 1190 Austria; ^2^ Institute for Arctic and Marine Biology UiT‐The Arctic University of Norway Tromsø 9037 Norway

**Keywords:** alpine–nival ecotone, climate change impact indicator, GLORIA, high mountain plants, long‐term monitoring, species composition change, species richness, thermophilisation

## Abstract

- High mountain ecosystems and their biota are governed by low‐temperature conditions and thus can be used as indicators for climate warming impacts on natural ecosystems, provided that long‐term data exist.
- We used data from the largest alpine to nival permanent plot site in the Alps, established in the frame of the *Global Observation Research Initiative in Alpine Environments* (GLORIA) on Schrankogel in the Tyrolean Alps, Austria, in 1994, and resurveyed in 2004 and 2014.
- Vascular plant species richness per plot increased over the entire period, albeit to a lesser extent in the second decade, because disappearance events increased markedly in the latter period. Although presence/absence data could only marginally explain range shift dynamics, changes in species cover and plant community composition indicate an accelerating transformation towards a more warmth‐demanding and more drought‐adapted vegetation, which is strongest at the lowest, least rugged subsite.
- Divergent responses of vertical distribution groups of species suggest that direct warming effects, rather than competitive displacement, are the primary causes of the observed patterns. The continued decrease in cryophilic species could imply that trailing edge dynamics proceed more rapidly than successful colonisation, which would favour a period of accelerated species declines.

## Introduction

High mountain plants are adapted to low‐temperature conditions (Körner & Larcher, 1988) and, apart from low latitudes, to a short growing season, which makes them sensitive to increasingly warmer climates. Alpine and subnival plants, however, may respond only little to short‐term climatic oscillations, but rather to longer lasting climatic trends, because most species are persistent, slow growing and long lived; annual and short‐lived species are rare above the treeline (Billings & Mooney, 1968; Körner, 2003; de Witte & Stöcklin, 2010). High mountain ecosystems, especially above the alpine grassland zone, are governed by climatic factors, whereas the importance of biotic factors, such as competition among species for light and nutrient resources and direct human impacts, for example through farming and livestock grazing, decreases with elevation. Therefore, changes in the occurrence of alpine and subnival plant species and in the composition of their assemblages are highly relevant as indicators of ecological impacts of climate change (Theurillat & Guisan, 2001; Grabherr *et al*., 2010; Malanson *et al*., 2011).

The last three decades were globally the warmest on record and, in the northern hemisphere, the period from 1983 to 2012 was probably the warmest of the last 1400 years (Hartmann *et al*., 2013; Luterbacher *et al*., 2013). Moreover, global climate warming tends to amplify in high‐elevation areas, compared with lowland areas (Barry, 2008; Ohmura, 2012; Mountain Research Initiative EDW Working Group, 2015), with a *c*. 1.2 times faster rise in annual mean temperatures at high‐elevation stations (> 500 m above sea level, asl) over the period 1961–2010 (Wang *et al*., 2016). Across the European Alps, high‐elevation stations show uniform warming trends of 0.8°C annual mean between 1981 and 2010, and 2.5°C mean from April to June, which is 3.5 times larger than the corresponding northern hemisphere temperature rise (Marty & Meister, 2012). Changes in precipitation show a larger regional and seasonal variability, especially in Europe (Kovats *et al*., 2014). Nevertheless, increases in evaporation and atmospheric humidity, as well as reductions in snow amount and snowpack period, are corresponding consequences of climate warming (Jiménez Cisneros *et al*., 2014). Although the spatial patterns of snow are determined by topography and the prevailing wind direction, the temporal patterns of snowmelt are directly linked to temperature change (Friedel, 1961; Kirkpatrick *et al*., 2017), and snow cover duration in the Alps showed a declining trend during the last decades (Gottfried *et al*., 2011; Cramer *et al*., 2014). Future scenarios predict a continued shift from snow to rain in mountainous regions, which alone could lead to a significant decrease in snow cover duration in central Europe (Steger *et al*., 2013; Jiménez Cisneros *et al*., 2014). This would result in an increase in the length of the growing seasons, and hence in a potential threat to high‐elevation plant species through the opening of immigration pathways for competitors from lower elevations (Dullinger *et al*., 2007; Steger *et al*., 2013). Increased drought risk has already been detected in central Europe over the past century, with surface warming as the primary cause after the mid‐1980s, and can be expected for the region of the Alps in the future (Dai *et al*., 2004; Gobiet *et al*., 2014). The climatically suitable area of alpine habitats is therefore successively shrinking. Depending on the climate change scenario, model projections suggest a loss of > 80% of habitats in some European mountains, including parts of the Alps, for up to 55% of the alpine species studied, until the end of the century (Engler *et al*., 2011).

Habitat loss, however, may not be immediately accompanied by a rapid species decline, which could lag behind for several decades, because of the long‐lived nature of most alpine plants (Dullinger *et al*., 2012b). A topographically diverse habitat situation may further buffer against the loss of climatically suitable habitats (Scherrer & Körner, 2011; Opedal *et al*., 2015). Upwardly advancing treelines, however, have been repeatedly observed (Kullman, 2002; Harsch *et al*., 2009; Hagedorn *et al*., 2014), as well as increasing numbers of vascular plant species at alpine to nival sites (Grabherr *et al*., 1994, 2001; Bahn & Körner, 2003; Klanderud & Birks, 2003; Holzinger *et al*., 2008; Vittoz *et al*., 2008; Stöckli *et al*., 2011; Pauli *et al*., 2012; Wipf *et al*., 2013; Steinbauer *et al*., 2018). Among the colonising species, an over‐representation of more warm‐demanding (i.e. thermophilic) species was found on summits distributed across the alpine life zone from the Mediterranean to boreal Europe (i.e. thermophilisation; Gottfried *et al*., 2012), which was primarily caused by an upward shift of plant species ranges (Pauli *et al*., 2012).

**Figure 1:**
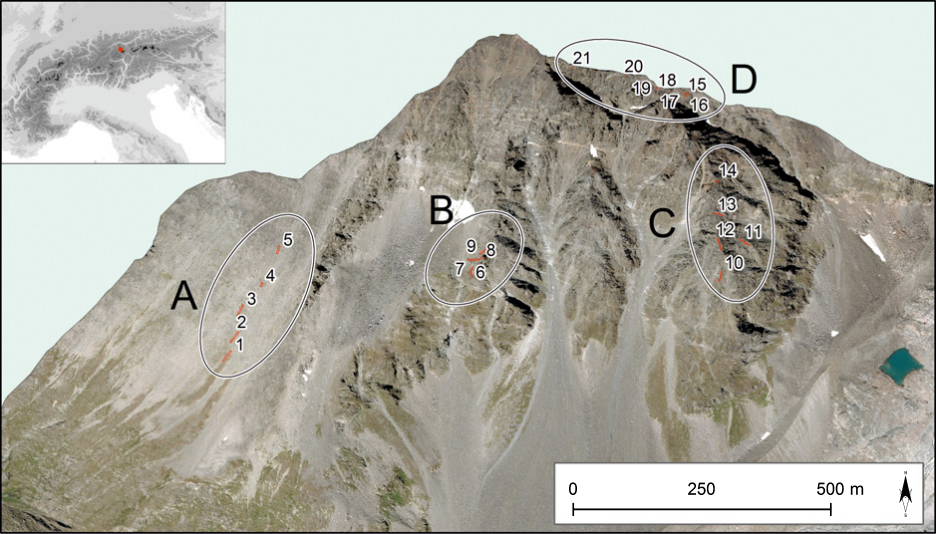
Location of permanent plots on Mount Schrankogel. Plots are grouped in transects clustered into four topographic blocks: (A) uniformly shaped southwest slope, rich in scree; (B) rugged south‐facing ridge; (C) rugged south–southeast‐facing ridge; and (D) south‐facing, high‐elevation plots along the east ridge. Orthophotos (© Land Tirol) modified with ArcGIS 10.3. for Desktop, Esri Inc. Details of transects are shown in Supporting Information Fig. S1.

In order to determine climate‐driven changes in species distribution, an extensive setting of permanent plot transects was established in 1994 across the alpine–nival ecotone of Schrankogel in the central Tyrolean Alps as part of the *Global Observation Research Initiative in Alpine Environments* (GLORIA, http://www.gloria.ac.at; Pauli *et al*., 2015). This ecotone is the transition zone between closed alpine grassland and open subnival plant assemblages (Gottfried *et al*., 1998; Pauli *et al*., 1999). The elevation of the ecotone on Schrankogel was found to strongly coincide with that of the median summer snow duration (i.e. where the probability of snow cover is 50% during the period June to August) derived from data across the Alps (Gottfried *et al*., 2011). Changes in species composition can be expected to be discernible earlier at the alpine–nival ecotone, where the upper range limits of the more thermophilic alpine grassland species and the lower margins of cold‐adapted (i.e. cryophilic) subnival–nival species coincide (Gottfried *et al*., 1999, 2011). The GLORIA master site Schrankogel is the largest permanent plot site close to the elevation limits of vascular plant life in the Alps, with four spatially separated subsites (blocks), representing elevations and different topographic complexity of the mountain's southerly oriented slope system (Fig. 1). Resurveys were undertaken in 2004 and 2014, thus spanning the period of amplified anthropogenic climate warming (Böhm *et al*., 2001; Marty & Meister, 2012; Hartmann *et al*., 2013).

After the first decade, an increase in species richness, resulting from colonisations of species into the plots, but hardly any disappearances from the plots, was observed. The novel aspect, however, was the evidence of decreasing abundance of all subnival–nival, i.e. outstandingly cold‐adapted, species (Pauli *et al*., 2007). Through the third survey in 2014, we assess whether the observed changes in species distribution patterns constitute ongoing trends in relation to recent climate warming by addressing the following four topics and inherent hypotheses. (1) Changes in vascular plant species diversity: (1a) species richness continues to increase, but (1b) the ratio of colonisations vs disappearances shifts towards the latter. (2) Changes in vascular plant cover: (2a) the total cover of vascular plants is increasing because (2b) the continued expansion of more thermophilic alpine and alpine–subnival pioneer species exceeds the ongoing decline of cryophilic high‐elevation species. (3) Changes in community‐weighted ecological indicators: the composition of species and their abundances change directionally in relation to climate trends, resulting in (3a) a thermophilisation of plant communities (Gottfried *et al*., 2012) and (3b) a more drought‐tolerant species composition. (4) Topography and elevation: plots in rugged habitats and in high elevations show lower rates of change, because habitat complexity in rocky terrain as well as low‐temperature conditions buffer against colonisation events and the expansion of established species.

## Materials and Methods

### Study area and design

The GLORIA master site Schrankogel (3497 m) is located in the Stubaier Alps, Tyrol, Austria. The bedrock mainly consists of gneiss (Hammer *et al*., 1929; Purtscheller, 1978); typical soil types are leptosols and cambisols (Hofmann *et al*., 2016). Characteristic plant communities of the upper alpine zone are grassland with *Carex curvula* and *Oreochloa disticha* (Caricion curvulae) and subnival to nival plant assemblages on siliceous screes (Androsacion alpinae; Grabherr, 1993; Abrate, 1998; Dullinger, 1998). Schrankogel is part of the protected area ‘Ruhegebiet Stubaier Alpen’, ranked in the International Union for Conservation of Nature (IUCN) category IV (UNEP‐WCMC & IUCN, 2018), and of the Long‐Term Socio‐economic and Ecosystem Research (LTSER) platform Tyrolean Alps (Mirtl *et al*., 2015).

In 1994, *c*. 1000 plots of 1 × 1 m^2^ were established as permanent plots across and above the alpine–nival ecotone (2911–3457 m), arranged in transects in order to cover the main habitat types of the alpine–nival ecotone of Schrankogel's southwest‐ to southeast‐facing slope system (Gottfried *et al*., 1998). Transects were grouped into four blocks (A, B, C and D), where (A) encompasses the uniformly shaped southwest slope which extends uninterruptedly from the alpine grassland belt to the alpine–nival ecotone, (B) encompasses the rugged south‐facing middle, (C) encompasses the rugged south–southeast‐facing eastern part within the ecotone and (D) encompasses the south‐facing, high‐elevation outposts in the nival zone along the east ridge (Fig. 1, Supporting Information Fig. S1; Table S1). The steep, mostly unvegetated, northern face and gullies and cliffs on the southern side had to be excluded, because of inaccessibility, unstable material and a high rockfall frequency.

In 2004, a representative subset of 362 plots was resurveyed in order to include all predominant plant communities (Pauli *et al*., 2007) distinguished by Pauli *et al*. (1999) and, in 2014, a larger subset of 661 plots was reinvestigated.

In each of the three survey campaigns, all vascular plant species were recorded and the percentage cover of each vascular plant species was estimated visually in each plot. At both resurvey campaigns, only data recorded without the aid of previous survey data were used.

### Data analyses

After removing plots in which disturbances (through rockfalls and substrate movements) had occurred, the dataset involving all three surveys (1994–2004–2014; Table S1) included 355 plots. A second dataset was constructed, only involving the first and third surveys (1994–2014), with a total of 654 plots (results are shown only in Supporting Information). Two annual species (*Euphrasia minima* and *Gentianella tenella*; Table S2) were removed from the datasets because of high inter‐annual fluctuation, which can strongly influence colonisation and disappearance rates.

Analyses were carried out using the entire dataset and also separately for each topographic block (A, B, C and D). As a result of the spatial arrangement of plots in transects, clustered in blocks, and the temporal dimension of the data (resurveys of the same plots at decadal intervals), all statistical models included a random intercept term with the structure: plot nested in transect (except for block D, where transects consisted of too few plots; Table S1), nested in block (the latter only for analyses over the entire study area).

All statistical analyses were performed in R v.3.1.3 (R Core Team, 2015). The significance of the effects of the predictor variables of all models was tested with the lsmeans function; for pairwise comparison, the cld function was used (package lsmeans; Tukey's honestly significant difference (HSD); Lenth, 2016). Table 1 gives an overview of the statistical models employed and the hypotheses they address.

**Table 1:** Overview of the statistical models employed

Hypotheses	Response	Fixed effects	Random effects	Model type (function)	Error distribution	Results shown in
1, 4	Species richness per plot	Year	Block/transect/plot	GLMM (glmmPQL)	Poisson	Fig. 2(a); Supporting Information Tables S4, S5
1	Species richness per plot and AR	Year × AR	Block/transect/plot	GLMM (glmmPQL)	Negative binomial	Fig. S3(b); Tables S6, S7
1, 4	Number of colonisation or disappearance events per plot	Decade × Type (Colon. or Disapp.)	Block/transect/plot	GLMM (glmer)	Negative binomial	Fig. 2(b); Tables S8, S9
1	Colonisation success per AR	Decade × AR	Block/transect/plot, Species	GLMM (glmmadmb)	Binomial	Fig. S4(a); Tables S10, S11
1	Disappearance success per AR	Decade × AR	Block/transect/plot, Species	GLMM (glmmadmb)	Binomial	Fig. S4(b); Tables S10, S11
2, 4	Cover sum per plot	Year	Block/transect/plot	LMM (lmer)	Gaussian	Fig. 3; Tables S12, S13
2	Cover sum per species and AR	Year × AR	Species	LMM (lmer)	Gaussian	Fig. S5(b); Tables S14, S15
3, 4	Thermic indicator per plot	Year	Block/transect/plot	LMM (lmer)	Gaussian	Fig. 4(a); Tables S16–S18
3, 4	Soil moisture indicator per plot	Year	Block/transect/plot	LMM (lmer)	Gaussian	Fig. 4(b); Tables S19–S21
4	Topographic differences among blocks	Block	Transect	LMM (lmer)	Gaussian	Figs S7, S8; Table S22

Given are the hypotheses addressed, model type (GLMM, generalised linear mixed‐effects model; LMM, linear mixed‐effects model) and corresponding R function used, error distribution, response variables, fixed and random effects, and figures and tables in which the results are shown. AR, altitudinal rank.

### Changes in vascular plant species diversity

For the response variables species richness, colonisation and disappearance, here treated as counts, the appropriate distribution for the models was determined by building two generalised mixed‐effects models (GLMMs), one assuming a Poisson distribution and one assuming a negative binomial distribution, which better fits many zero values (zero inflation; glmer and glmer.nb from lme4 package, respectively; Bates *et al*., 2015). A likelihood ratio test between these two models was performed (ANOVA function from stats package) to determine the appropriate model in each case (*P* < 0.05). For both models, over‐dispersion was tested with the overdisp.glmer function from the RVAideMemoire package (Hervé, 2016). In cases of over‐dispersion, the function glmmPQL from the mass package (Venables & Ripley, 2002) was used, which employs penalised quasi‐likelihood and takes an over‐dispersion parameter into account.

Species richness was calculated as the number of species per plot and survey, and was analysed as Poisson distributed counts with year (i.e. survey) as predictor. Species richness data within the blocks were over‐dispersed (over‐dispersion parameter of the glmer model > 1.2), and therefore the function glmmPQL was used.

Colonisations were defined as the number of species per plot present at the time of the resurvey, which were absent in the respective plot at the previous survey, and disappearances were defined as the number of species per plot absent at the time of the resurvey, which were present at the previous survey. These counts were analysed with GLMMs with a negative binomial distribution, with type (colonisation or disappearance) and decade as fixed effects.

As a proxy for the thermal preferences of species, species with different distributions along the elevation gradient were assigned to species groups of different altitudinal ranks (ARs) after Gottfried *et al*. (2012) (Table S3). To determine which AR species group drives the observed changes, species richness and relative colonisation and disappearance events (the proportion of the number of plots not yet occupied in the case of colonisation, and of previously occupied plots in the case of disappearance) were calculated for each AR separately, and the above analyses on species richness were repeated with AR included in the fixed effects. Colonisation and disappearance events were treated as Bernoulli trials (1, successful colonisation of an empty plot or disappearance from an occupied plot of a given species; 0, plot not colonised or species not disappeared from a plot) and modelled as binomial GLMMs (function glmmadmb in package glmmADMB; Fournier *et al*., 2012).

### Changes in vascular plant cover

Changes in cover sums (i.e. cumulative cover of all species present in a plot) were modelled using linear mixed‐effect models (LMMs, function lmer of package lme4) with survey year as the only fixed effect, and, additionally, with AR as another fixed effect.

### Changes in community‐weighted ecological indicators

To investigate directional changes in plant species composition, the following ecological indicator values of the species occurring in a plot were used: AR (Table S3) and the soil moisture indicator values in Landolt *et al*. (2010), which were available for all species. All other available indicators by Landolt *et al*. (2010) were not present in a sufficient dispersion along their gradients to enable a reasonable statistical analysis (e.g. temperature T), did not indicate any significant effects (e.g. continentality K, nutrients N) or were not meaningful in the context of this study (e.g. soil reaction R). Each rank was given a number which represents the position along an environmental gradient; for AR: 1 = subnival–nival, 2 = alpine–subnival, 3 = alpine, 4 = (montane–)treeline–alpine species; for soil moisture: 1 = very dry to 4 = very moist. The thermic and soil moisture indicators were calculated after Gottfried *et al*. (2012) for each plot, and survey as an averaged composite score of the AR and the soil moisture indicator values, respectively, weighted by the cover of the occurring species:

$$\begin{array}{c} \text{Indicator}=(\sum \text{rank}\left({\text{species}}_{i}\right)\times \text{cover}\left({\text{species}}_{i}\right))/\\ \sum \text{cover}({\text{species}}_{i}) \end{array}$$

The thermic indicator was significantly positively correlated with temperature sums derived from *in situ* soil temperature measurements (Fig. S2). Each indicator was analysed after tests for normal distribution with survey year as fixed effect with LMMs. To indicate any direction of changes in community‐weighted ecological indicators between surveys, the effect size of the predictor year and its associated confidence interval were used as Δ indicator:

$$\Delta \;\text{indicator}={\text{Indicator}}_{t+1}-{\text{Indicator}}_{t}$$

The correlation between Δ thermic indicator and Δ soil moisture indicator per plot was modelled using LMMs with plot (nested in transect nested in block) and decades as random intercept terms.

### Topography and elevation

To assess the topographic similarity among the four blocks, non‐metric multidimensional scaling (NMDS) was used (function metaMDS, R package vegan; Oksanen *et al*., 2018). A matrix was built with rescaled parameters for each plot, that is, estimated top cover of surface types (rock, scree, bare soil and vegetation) and topographic parameters (altitude, aspect, slope and ruggedness) derived from a digital elevation model (© Land Tirol). Ruggedness was calculated as the standard deviation of elevation with a 100 × 100 m^2^ raster per plot using ArcGIS 10.3. for Desktop, Esri Inc. Plotting was performed using the package ggplot (Wickham, 2009). To analyse differences among blocks, an LMM with the first NMDS axis as response, block as fixed effect and transect as random effect was fitted.

## Results

**Figure 2:**
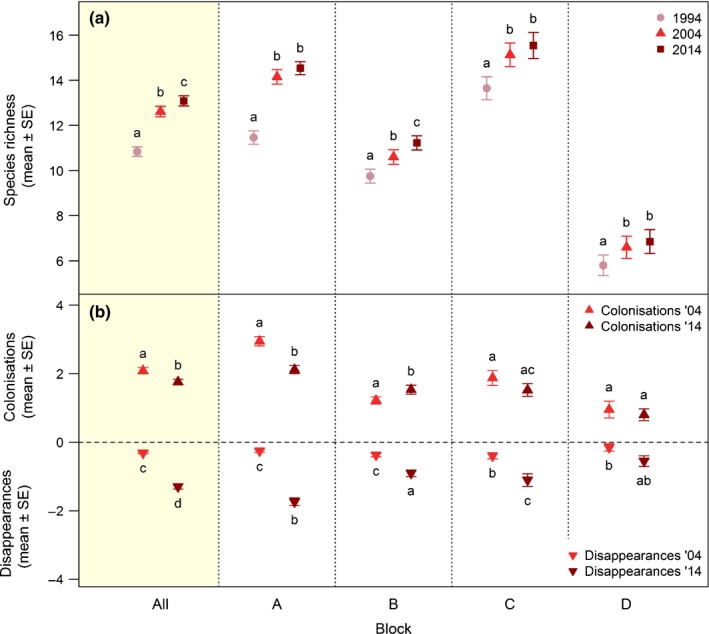
Changes in vascular plant species diversity on Mount Schrankogel. Mean ± SE of raw data of (a) species richness (Supporting Information Table S4) and (b) numbers of colonising and disappearing species (Table S8) per plot on Mount Schrankogel in the survey years 1994, 2004 and 2014. Mean values over the entire study area (All, shaded) and for each block (A, B, C, D) are shown. For plot numbers per block, see Table S1. Different lowercase letters denote significant differences: (a) between the survey years within each block based on generalised mixed‐effects models using penalised quasi‐likelihood with a negative binomial distribution (Table S5); and (b) between the survey years and types (colonisations, disappearances) within each block based on generalised mixed‐effects models with a negative binomial distribution (Table S9).

**Figure 3:**
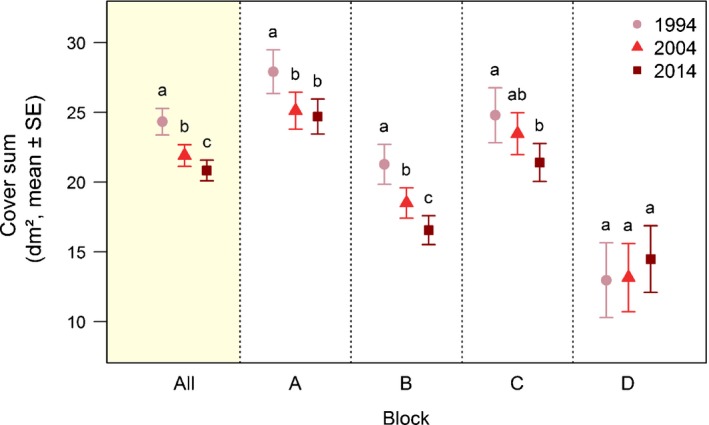
Changes in vegetation cover of vascular plants on Mount Schrankogel. Mean ± SE of raw data of cover sum (Supporting Information Table S12) per plot on Mount Schrankogel in the survey years 1994, 2004 and 2014. Mean values over the entire study area (All, shaded) and for each block (A, B, C, D) are shown. For plot numbers per block, see Table S1. Different lowercase letters denote significant differences between the survey years within each block based on linear mixed‐effects models (Table S13).

**Figure 4:**
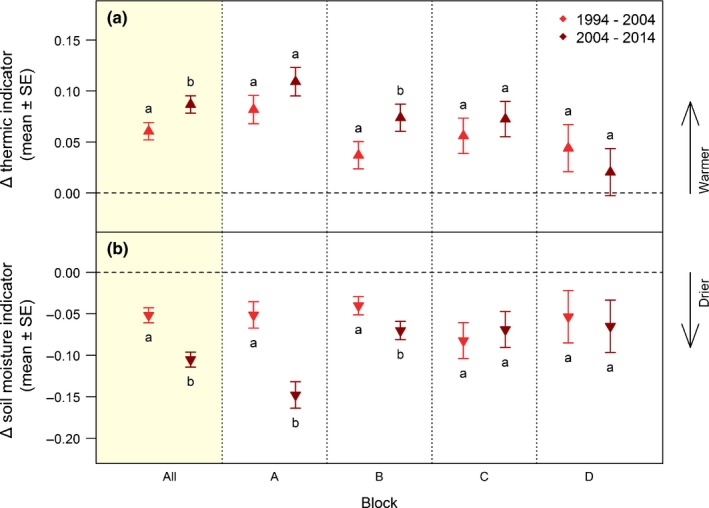
Changes in community‐weighted ecological indicators. Changes in (a) thermic indicator (Gottfried *et al*., 2012) and (b) soil moisture indicator in the periods 1994–2004 and 2004–2014 (Supporting Information Tables S16, S19). Modelled effect sizes and ± SE over the entire study area (All, shaded) and for each block (A, B, C, D) are shown (Tables S17, S20). For plot numbers per block, see Table S1. Different lowercase letters denote significant differences between the changes in indicators from the first to the second decade within each block based on linear mixed‐effects models (Tables S18, S21).

Changes in species richness, colonisation, disappearances and community‐weighted ecological indicators showed equal trends in dataset 1994–2004–2014 (involving three surveys with 355 plots, see below) and 1994–2014 (involving only the first and third surveys with 654 plots). Therefore, only the former is reported here (Figs 2, 3, 4, S3–S8; Tables S4–S22) and the latter is provided in Supporting Information (Tables S23–S30).

### (1) Changes in vascular plant species diversity

### Species richness

The total number of species increased from 51 species in 1994 to 54 in 2004 and 61 in 2014.

The mean species number per plot increased from 10.84 species in 1994 to 12.61 in 2004 and 13.08 in 2014 (Fig. 2a; Table S4), and was significantly higher at the later surveys than at the preceding ones (GLMMs, *P* < 0.001 in all cases; Table S5).

Within ARs, the total number of species was stable with six and 20 species in each survey at AR1 and AR2, respectively, whereas species numbers increased slightly in AR3 (18, 19 and 20 species in 1994, 2004 and 2014, respectively), and more than doubled in AR4 between 1994 and 2014 (six, eight and 13 species; Table S3). Species richness summed over all plots (Fig. S3a) and mean species numbers per plot (Fig. S3b) were highest in AR2, followed by AR1, and increased significantly only between 1994 and 2004 within AR2 and AR3 (GLMMs, *P* < 0.0001; Tables S6, S7).

#### Colonisations and disappearances

The mean number of colonisations decreased significantly from 2.08 species per plot between 1994 and 2004 to 1.76 between 2004 and 2014. The mean number of disappearances increased significantly from 0.31 species per plot in the first decade to 1.29 in the second decade (means calculated from raw data; effect sizes from GLMMs, *P* < 0.05; Fig. 2b; Tables S8, S9). No species, however, disappeared completely from the whole area. By contrast, four and seven species were recorded for the first time in 2004 and 2014, respectively (Fig. S3). These new species belonged exclusively to AR3 (one and two new species in 2004 and 2014, respectively) and AR4 (three and five species). Colonisation in relation to previously unoccupied plots was highest among AR1 species (Fig. S4a; Table S10), but decreased significantly from the first to the second decade (GLMMs, *P* = 0.01; Table S11), whereas there was no change among AR2–4 species. Relative disappearance was fairly low (2–3%) in AR1–3 in the first decade, whereas AR4 species disappeared from almost a quarter of their formerly occupied plots (Fig. S4b; Table S10). In the second decade, however, relative disappearance increased sharply and significantly by factors of 3.23, 2.65 and 6.9 in species of AR1–3, respectively, whereas AR4 remained stable (GLMMs; Table S11).

Exotic species and woody plants did not occur in or colonise the permanent plots during the study period.

### (2) Changes in vascular plant cover

(2a) The mean cover sum of all vascular plant species per plot decreased significantly from 24.33 dm² in 1994 to 21.9 dm² and 20.83 dm² in 2004 and 2014, respectively (LMMs, *P* < 0.05; Fig. 3; Tables S12, S13).

(2b) Cover sums over all plots for each AR were initially highest in AR1 and AR2, followed by AR3 and AR4 (Fig. S5a). Over time, cover sums of AR1 showed a pronounced linear decrease, whereas those of AR2 increased. The mean cover sums per species were highest in AR1 in all 3 years, but decreased significantly over time, whereas there was no change in the other ARs (GLMMs; Fig. S5b; Tables S14, S15).

### (3) Changes in community‐weighted ecological indicators

#### Thermic indicator of plant communities

The thermic indicator increased significantly over successive surveys from 1.61 in 1994 to 1.67 in 2004 and 1.76 in 2014, with a Δ thermic indicator of 0.06 in the first decade and 0.09 in the second decade (GLMMs, *P* < 0.001; Fig. 4a; Tables S16–S18).

#### Soil moisture indicator of plant communities

The soil moisture indicator decreased significantly over successive surveys from 2.95 in 1994 to 2.89 in 2004 and 2.79 in 2014, with a Δ soil moisture indicator of −0.05 for the first decade and −0.11 for the second decade (GLMMs, *P* < 0.001; Fig. 4b; Tables S19–S21).

ARs and the Landolt soil moisture indicator were not correlated at the species level (Spearman rank correlation, rho = −0.196, *P* = 0.134). At the plot level, however, Δ soil moisture indicator decreased significantly with increasing Δ thermic indicator (LMM, *P* < 0.0001; Fig. S6).

### (4) Topography and elevation

Plots in the lowest block A had, on average, the largest proportion of scree and vegetation cover, and the lowest proportion of solid rock (Fig. S7). Further, they showed the lowest degree of ruggedness and steepness. The nival, that is, highest, block D was the most rugged and steepest block. The topographic parameters (first axis of NMDS; Fig. S8) of block A differed significantly from those of blocks B and D; block D differed significantly from all other blocks, whereas blocks B and C were not significantly different (Table S22).

Changes in species richness in individual blocks showed the same tendencies as in the entire dataset (Fig. 2a; Table S4), even though the increase in richness stagnated in all blocks, except in block B in the second decade (Table S5). The number of colonisations decreased significantly in block A as over the whole study area, whereas there was no significant change in blocks C and D, and, in block B, colonisations even increased in the second decade. The number of disappearances increased significantly in all blocks as in the entire dataset, except in block D, where no significant change was observed (Fig. 2b; Tables S8, S9).

Vascular plant cover decreased significantly over time in all blocks, except D, where no significant change in cover sums was observed. In block A, however, the decrease stopped in the second decade (Fig. 3; Tables S12, S13).

Changes in community‐weighted ecological indicators per block did not fundamentally deviate from the results over all plots. A significant overall increase in the thermic indicator, however, was only reached in block B (Fig. 4a; Tables S16–S18), whereas a significant overall decrease in the soil moisture indicator was also found in blocks A and B (Fig. 4b; Tables S19–S21).

## Discussion

Over a decade ago, the first repeated survey of the 10‐yr‐old permanent plots in the alpine–nival ecotone of Schrankogel showed an increase in species numbers similar to other studies, but, most noteworthy, also a divergent change in species abundance, providing the first consistent evidence of population declines of cryophilic species (Pauli *et al*., 2007). After 20 yr of progressing climate warming, we find that (1) species turnover involves the disappearances of species almost exclusively in the second decade, (2) the decrease in vegetation cover constitutes an ongoing trend over both decades, (3) the plant community transformation towards more thermophilous species assemblages, which are increasingly adapted to drier soil conditions, has accelerated, and (4) differences among the blocks reflect the temperature gradient, topography and connectivity to lower elevation species pools.

### Changes in vascular plant species diversity

The prevailing net gain in vascular plant species richness found on Schrankogel (Fig. 2a) confirms the ample evidence from other parts of the Alps (Grabherr *et al*., 1994; Stöckli *et al*., 2011; Matteodo *et al*., 2013), temperate to boreal mountains across Europe (Britton *et al*., 2009; Pauli *et al*., 2012; Grytnes *et al*., 2014; Steinbauer *et al*., 2018) and in parts of temperate–continental North America (Lesica, 2014). There is little doubt that the increase in species numbers has been caused by an upward shift of species previously occurring at lower elevations (Odland *et al*., 2010; Pauli *et al*., 2012). Range shifts towards higher elevation are commonly driven by climate warming (Chen *et al*., 2011), which has been pronounced in the Alps during recent decades (Marty & Meister, 2012), whereas stagnating species numbers over a 14‐yr period of rather stable temperature conditions have been found in southern Norway (Vanneste *et al*., 2017). In the Alps, increases in species numbers have even been observed to accelerate at century‐old study sites on high‐alpine to nival summits during recent decades (Walther *et al*., 2005; Wipf *et al*., 2013; Steinbauer *et al*., 2018).

The increase in species richness in our plots was smaller in the second decade (Fig. 2a), suggesting a slowing down of species upward shifts. The separate consideration of colonisation and disappearance of species, however, showed that the number of colonisations decreased only slightly, whereas the number of disappearances increased markedly (Fig. 2b). Species disappearances at the alpine–nival ecotone can indicate retracting lower range margins, as expected through warming‐driven competitive displacements (Engler *et al*., 2011; Lenoir & Svenning, 2013). In particular, cold‐adapted species have been found recently to have experienced range contractions in the Alps (Rumpf *et al*., 2018) and alpine Mediterranean species have been shown to decline, possibly as a result of the combined effects of warming and a reduction in precipitation (Pauli *et al*., 2012). Species disappearances on Schrankogel, however, did not only concern subnival–nival species (AR1), but also alpine–subnival pioneer species (AR2) and alpine species (AR3) in similar proportions (Fig. S4b). Species colonisation numbers remained stable in all altitudinal species groups, except for AR1, where they dropped significantly (Fig. S4a). We therefore cannot unequivocally attribute the observed species turnover to warming‐driven range dynamics. The detection of leading edge shifts driven by climate warming is hampered by the stochastic nature of processes, such as the propagation of diaspores, germination and establishment of seedlings. Similarly, the disappearance of species may either be the final stage of a population decline or the result of an unsuccessful species establishment, which are difficult to disentangle (Grytnes *et al*., 2014). Further, projected directional range shifts and associated local species extinctions in temperate and boreal mountains (Engler *et al*., 2011) may still require longer periods for the usually long‐lived perennial alpine plants. Species dwelling in cold environments may persist even in climatically unsuitable habitats, and thereby accumulate an extinction debt (Dullinger *et al*., 2012a). Yet, the increase in richness in our plots was mainly driven by alpine–subnival pioneer species (AR2), whereas the number of subnival–nival species (AR1) decreased slightly (Fig. S3), which could already be an indication of warming‐driven range shift dynamics. This also accounts for the fact that newly appearing species all belong to the lower elevation groups (AR3 and AR4; Table S3), which is in line with increasingly rising species numbers on mountain summits in Europe (Steinbauer *et al*., 2018).

### Changes in vascular plant cover

In contrast with increasing species numbers, cover sums of vascular plant species showed a decreasing trend (Fig. 3). This was mainly driven by a strong continued population decline of all subnival–nival species (AR1), which could not be compensated by the ongoing increase in alpine–subnival species (AR2; Fig. S5).

The strongly divergent cover change of altitudinal species groups clearly depicts a shift in habitat suitability at the range margins of species, and is consistent with Cotto *et al*. (2017) and Rumpf *et al*. (2018), suggesting that population declines are occurring more rapidly than range shifts. This could lead, at least transitionally, to a disruption of distribution patterns, rather than to a rapid greening of the alpine–nival ecotone. The importance of directional changes in species cover was also shown in alpine permanent plots in the Montana Rocky Mountains by Lesica (2014), who noted that changes in species abundance can reveal far more sensitive responses to climate change effects than presence/absence data.

The most relevant potential mechanisms underlying the observed patterns are, first, a successive competitive displacement of the high‐elevation species (AR1) through expansion of lower elevation species (AR2, AR3) and, second, direct climatic effects which may deteriorate the performance of cryophilic species. Competition effects, as were verified experimentally (Elmendorf *et al*., 2012; Alexander *et al*., 2015), are potentially relevant; however, conspicuous signs of competition pressure, such as for light through taller growing species, are not common at the alpine–nival ecotone, as all species are dwarf‐stature plants. Further, the typical habitats at the alpine–nival ecotone do not have a closed vegetation cover (*c*. 10–25% of the plot surface; Fig. S7). Although species of different elevational distribution preferences can grow in the direct neighbourhood, habitats at the alpine–nival ecotone are governed by abiotic, mostly climatic, factors, where the stress gradient hypothesis (Bertness & Callaway, 1994; Callaway *et al*., 2002) would posit that facilitative interspecific interactions outweigh competitive effects.

Overall, however, vascular plant cover is decreasing, which suggests that advancing alpine–subnival species (AR2) cannot fill the space released by AR1 species. We therefore assume that direct climatic effects may be of superior relevance for an increasing maladaptation and population decline of cryophilic species. Ecophysiological studies of cryophilic species have not been conducted often, but some species, including several of our AR1 group, have been found to show high heat sensitivity and low ability to acclimate respiration rates to higher temperatures, causing detrimentally high respiration rates, and thus the plants rapidly attain a negative carbon balance (Larigauderie & Körner, 1995; Larcher *et al*., 1997; Cooper, 2004). Such metabolic disadvantages may explain the continued population decline even in the absence of competition through higher temperatures alone. Drier conditions, through earlier snowmelt and stronger evapotranspiration, can further deteriorate the situation by leading to lower soil moisture levels, and thus to warmer soils, which could cause detrimentally high root respiration rates (Cooper, 2004; Lesica, 2014).

### Changes in community‐weighted ecological indicators

Changes in plant community composition in our plots show a transformation towards a more thermophilic vegetation in both decades (Fig. 4a), in congruence with Gottfried *et al*. (2012). Most noteworthy, the thermophilisation signal, already significant in the first decade of observation 1994–2004 (Table S17), was significantly stronger during the second decade 2004–2014 (Table S18).

Thermophilisation effects have substantial consequences for subnival plant communities in the alpine–nival ecotone, where AR1 species have the centre of their distribution close to their rear edge (Gottfried *et al*., 1999). This ecotone was found to strongly coincide with the altitude of the summer snow line (Gottfried *et al*., 2011). Plant assemblages at the ecotone should therefore respond sensitively to warming and associated shifts in temporal patterns of snow duration. High‐altitude weather stations distributed over the central and northern Alps show a uniform temperature increase in annual mean temperatures of 0.8°C during the last three decades (Marty & Meister, 2012), which would correspond to 0.53°C in the period 1994–2014. Using the environmental lapse rate of −0.65°C per 100 m elevation, a temperature increase of 0.53°C corresponds to a difference of *c*. 80 m in elevation. As an approximation, one unit of the thermic indicator translates roughly to the elevation range of an entire vegetation belt, for example, the alpine belt on Schrankogel ranges from *c*. 2300 to 2800 m (Dullinger, 1998). The observed thermophilisation values of 0.06, 0.09 and 0.15 units during the first and second decade, and the 20‐yr period, respectively, thus approximate to 6%, 9% and 15% of a vegetation belt. This is within the same magnitude of change as observed by Gottfried *et al*. (2012) on the European level (i.e. 5% of one vegetation belt after 7 yr). Given a vegetation belt of a vertical extent of 500 m, the observed change of 0.15 units in the thermic vegetation indicator corresponds to 75 m in elevation. Thus, the vegetation at the alpine–nival ecotone seems to be largely tracking recent climate warming.

An even stronger amplification effect was detected for the soil moisture indicator (Table S21). Our results show a shift of the species composition towards more drought‐tolerant plant compositions, that is, an aridisation (Fig. 4b). Delta values of the community‐level soil moisture and thermic indicators were highly negatively correlated (Fig. S6), and hence mainly reflect changes in the same species. Yet, this does not necessarily mean that all species experiencing a disadvantage through warmer conditions effectively suffer from drought stress.

Higher temperatures, however, cause greater evapotranspiration, leading to reduced water availability, which alone can increase the probability of drought stress (Beniston, 2003). Regional scenarios on precipitation change are inconsistent; however, projections for central Europe show that mean precipitation tends to decrease in summer and increase in winter (Schmidli *et al*., 2007), with increasingly more rain instead of snow in mountainous regions (Steger *et al*., 2013). Snow cover is generally declining in the Alps, although patterns of changes in the amount of snowfall are also rather patchy (Gobiet *et al*., 2014). Moreover, the significant trend towards a longer annual snow‐free period has been shown to be consistent with reported trends of longer growing seasons (Dye, 2002; Giménez‐Benavides *et al*., 2007). Combined effects of higher temperatures and concomitant drier conditions can result in strong transforming forces on cold‐adapted plant communities (Lesica, 2014), which was also experimentally confirmed (De Boeck *et al*., 2016).

### Topography and elevation

Despite the deviating habitat situations among the four spatially separated blocks (Figs S7, S8), which may lead to different response patterns (Scherrer & Körner, 2011), changes in species occurrence, cover and composition were generally consistent, especially across the blocks in the ecotone (Figs 2, 3, 4). The observed deviations from the common trends, however, can contribute to a better understanding of possible causes and mechanisms behind the observed changes.

Contrary to the others, species patterns in the nival block D were rather stable over the 20‐yr period, which could be explained by the larger distance to the alpine species pool. Obviously, low‐temperature conditions suitable for cryophilic species still prevailed, that is, these populations occurred well above their lower range margin and above the upper margins of alpine species. Block D, however, consisted of fewer plots with fewer species, compared with the others. The results should therefore be treated with some caution.

The higher numbers of both colonising and disappearing species (Fig. 2b) and the stronger thermophilisation and aridisation signals (Fig. 4) at the uniform slope in the ecotone (block A) conform with the lowest elevation and closest connection to the alpine grassland zone. The different magnitudes of change among the four blocks reflect a gradient of increasing low‐temperature conditions and a thinning of species pools, and thus the importance of the particular position along the thermal gradient for the velocity of warming‐driven vegetation dynamics. This corresponds to recently observed effects over larger elevation ranges, where both species ranges and abundances changed more rapidly the lower a species was situated historically (Rumpf *et al*., 2018). In the alpine–nival ecotone on Schrankogel, vascular plant species cover decreased in general (Fig. 3). Interestingly, however, the cover remained stable in block A in the second decade, which could signal an early stage of infilling processes. Combined with the strong thermophilisation signal, this is likely to indicate an enhanced expansion of the more thermophilous species. By contrast, cover continued to decrease in the rugged blocks B and C, together with a significant thermophilisation (Table S17), and thus was mainly driven by the dieback of subnival–nival species. Rugged terrain did not restrain the decline of cryophilic species, but may have provided a barrier to the expansion of potential competitors. We therefore suggest that factors other than competition, such as direct temperature effects on plant metabolism, have strongly contributed to the decrease in cover of the cold‐adapted, high‐elevation species.

In conclusion, we argue that a combination of continued temperature rise and decreased snow cover duration has a major impact on the composition, performance and persistence of plant species in subnival communities. This is manifested by an accelerating loss of subnival plant communities in the central Alps. The longevity and persistence abilities of high‐elevation plants may have delayed the disappearance of species from habitats which have become climatically unsuitable (Dullinger *et al*., 2012a). Increasing maladaptation of cryophilic high‐elevation species to warmer and longer growing seasons, however, has led to their continued retraction, even in the absence of competitive displacement. An incomplete infilling through succeeding species from lower elevation further suggests that trailing edge dynamics proceed faster than leading edge advances in environments above the alpine grassland zone. If this holds true in further progress, it could imply that the pay‐off of a rising extinction debt (Kuussaari *et al*., 2009; Cotto *et al*., 2017) enters into force before colder habitats, if available, can be reached.

## Author contributions

H.P., M.W., A.L. and K.S. designed the study and were part of the recording team. P.R.S., A.L., K.S. and M.W. analysed the output data. H.P. managed the study. A.L., P.R.S. and H.P. wrote the manuscript. All authors discussed the results and implications, and commented on the manuscript at all stages. A.L. and K.S. contributed equally to this work.

## Supporting information

Please note: Wiley Blackwell are not responsible for the content or functionality of any Supporting Information supplied by the authors. Any queries (other than missing material) should be directed to the *New Phytologist* Central Office.

**Fig. S1** Location of permanent plots on Mount Schrankogel with transect details.
**Fig. S2** Correlation between thermic indicator and temperature sum on Mount Schrankogel.
**Fig. S3** Vascular plant species richness per altitudinal rank in the survey years 1994, 2004 and 2014.
**Fig. S4** Relative colonisation and disappearance per altitudinal rank in the periods 1994–2004 and 2004–2014.
**Fig. S5** Cover sum of vascular plant species per altitudinal rank in the survey years 1994, 2004 and 2014.
**Fig. S6** Correlation between the changes in thermic indicator and changes in soil moisture indicator per plot in the periods 1994–2004 and 2004–2014.
**Fig. S7** Topographic parameters per block on Mount Schrankogel.
**Fig. S8** Non‐metric multidimensional scaling (NMDS) of topographic parameters of plots.
**Table S1** Setup of permanent plots for the monitoring of vascular plant species on Mount Schrankogel
**Table S2** Frequency of annual plant species
**Table S3** Vascular plant species per altitudinal rank in the survey years 1994, 2004 and 2014
**Table S4** Vascular plant species richness in the survey years 1994, 2004 and 2014
**Table S5** Changes in vascular plant species richness in the periods 1994–2004, 1994–2014 and 2004–2014
**Table S6** Vascular plant species richness per altitudinal rank in the survey years 1994, 2004 and 2014
**Table S7** Changes in vascular plant species richness per altitudinal rank in the periods 1994–2004, 1994–2014 and 2004–2014
**Table S8** Number of colonising and disappearing species at the end of periods 1994–2004 and 2004–2014
**Table S9** Differences between numbers of colonising and disappearing species within and among the periods 1994–2004 and 2004–2014
**Table S10** Relative colonisation and disappearance per altitudinal rank in the periods 1994–2004 and 2004–2014
**Table S11** Changes in relative colonisation and disappearance per altitudinal rank in the periods 1994–2004 and 2004–2014
**Table S12** Cover sum of species in the survey years 1994, 2004 and 2014
**Table S13** Changes in cover sum of species in the periods 1994–2004, 1994–2014 and 2004–2014
**Table S14** Cover sum of vascular plant species per altitudinal rank in the survey years 1994, 2004 and 2014
**Table S15** Changes in mean cover sum of vascular plant species per altitudinal rank in the periods 1994–2004, 1994–2014 and 2004–2014
**Table S16** Thermic indicator in the survey years 1994, 2004 and 2014
**Table S17** Thermophilisation in the periods 1994–2004, 1994–2014 and 2004–2014
**Table S18** Changes in thermophilisation between the periods 1994–2004 and 2004–2014
**Table S19** Soil moisture indicator in the survey years 1994, 2004 and 2014
**Table S20** Change in soil moisture indicator in the periods 1994–2004, 1994–2014 and 2004–2014
**Table S21** Changes in Δ soil moisture indicator between the periods 1994–2004 and 2004–2014
**Table S22** Differences in abiotic factors between blocks on Mount Schrankogel
**Table S23** Vascular plant species richness in the survey years 1994 and 2014
**Table S24** Changes in vascular plant species richness in the period 1994–2014
**Table S25** Number of colonising and disappearing species in the period 1994–2014
**Table S26** Differences between numbers of colonising and disappearing species in the period 1994–2014
**Table S27** Thermic indicator in the survey years 1994 and 2014
**Table S28** Thermophilisation in the period 1994–2014
**Table S29** Soil moisture indicator in the survey years 1994 and 2014
**Table S30** Changes in soil moisture in the period 1994–2014

## References

[nph15290-bib-0001] Abrate S . 1998 Vegetationskarte des Schrankogel, Stubaier Alpen. Diplomarbeit, Universität Wien, Austria.

[nph15290-bib-0002] Alexander JM , Diez JM , Levine JM . 2015 Novel competitors shape species’ responses to climate change. Nature 525: 515–518. 26374998 10.1038/nature14952

[nph15290-bib-0003] Bahn M , Körner C . 2003 Recent increases in summit flora caused by warming in the Alps In: Nagy L, Grabherr G, Körner C, Thompson DBA, eds. Alpine biodiversity in Europe – a Europe‐wide assessment of biological richness and change. Berlin, Germany: Ecological studies 167, Springer, 437–441.

[nph15290-bib-0004] Barry RG . 2008 Mountain weather and climate, *3^rd^ edn* New York, NY, USA: Cambridge University Press.

[nph15290-bib-0005] Bates D , Maechler M , Bolker B , Walker S . 2015 Fitting linear mixed‐effects models using lme4. Journal of Statistical Software 67: 1–48.

[nph15290-bib-0006] Beniston M . 2003 Climatic change in mountain regions: a review of possible impacts. Climatic Change 59: 5–31.

[nph15290-bib-0007] Bertness MD , Callaway R . 1994 Positive interactions in communities. Trends in Ecology & Evolution 9: 191–193. 21236818 10.1016/0169-5347(94)90088-4

[nph15290-bib-0008] Billings WD , Mooney HA . 1968 The ecology of arctic and alpine plants. Biological Reviews of the Cambridge Philosophical Society 43: 481–529.

[nph15290-bib-0009] Böhm R , Auer I , Brunetti M , Maugeri M , Nanni T , Schöner W . 2001 Regional temperature variability in the European Alps: 1760–1998 from homogenized instrumental time series. International Journal of Climatology 21: 1779–1801.

[nph15290-bib-0010] Britton AJ , Beale CM , Towers W , Hewison RL . 2009 Biodiversity gains and losses: evidence for homogenisation of Scottish alpine vegetation. Biological Conservation 142: 1728–1739.

[nph15290-bib-0011] Callaway RM , Brooker RW , Choler P , Kikvidze Z , Lortie CJ , Michalet R , Paolini L , Pugnaire FI , Newingham B , Aschehoug ET *et al* 2002 Positive interactions among alpine plants increase with stress. Nature 417: 844–848. 12075350 10.1038/nature00812

[nph15290-bib-0012] Chen IC , Hill JK , Ohlemuller R , Roy DB , Thomas CD . 2011 Rapid range shifts of species associated with high levels of climate warming. Science 333: 1024–1026. 21852500 10.1126/science.1206432

[nph15290-bib-0013] Cooper EJ . 2004 Out of sight, out of mind: thermal acclimation of root respiration in Arctic *Ranunculus* . Arctic Antarctic and Alpine Research 36: 308–313.

[nph15290-bib-0014] Cotto O , Wessely J , Georges D , Klonner G , Schmid M , Dullinger S , Thuiller W , Guillaume F . 2017 A dynamic eco‐evolutionary model predicts slow response of alpine plants to climate warming. Nature Communications 8: 15399. 10.1038/ncomms15399 PMC5424169 28474676

[nph15290-bib-0015] Cramer W , Yohe GW , Auffhammer M , Huggel C , Molau U , da Silva Dias MAF , Solow A , Stone DA , Tibig L . 2014 Detection and attribution of observed impacts In: Field CB, Barros VR, Dokken DJ, Mach KJ, Mastrandrea MD, Bilir TE, Chatterjee M, Ebi KL, Estrada YO, Genova RC *et al*, eds. Climate Change 2014: impacts, adaptation, and vulnerability. Part A: global and sectoral aspects. Contribution of Working Group II to the Fifth Assessment Report of the Intergovernmental Panel on Climate Change. Cambridge, UK: Cambridge University Press.

[nph15290-bib-0016] Dai A , Trenberth KE , Qian TT . 2004 A global dataset of Palmer Drought Severity Index for 1870–2002: relationship with soil moisture and effects of surface warming. Journal of Hydrometeorology 5: 1117–1130.

[nph15290-bib-0017] De Boeck HJ , Bassin S , Verlinden M , Zeiter M , Hiltbrunner E . 2016 Simulated heat waves affected alpine grassland only in combination with drought. New Phytologist 209: 531–541. 26267066 10.1111/nph.13601

[nph15290-bib-0018] Dullinger S . 1998 Vegetation des Schrankogel, Stubaier Alpen. Diploma thesis, University of Vienna, Austria.

[nph15290-bib-0019] Dullinger S , Gattringer A , Thuiller W , Moser D , Zimmermann NE , Guisan A , Willner W , Plutzar C , Leitner M , Mang T *et al* 2012a Extinction debt of high‐mountain plants under twenty‐first‐century climate change. Nature Climate Change 2: 619–622.

[nph15290-bib-0020] Dullinger S , Kleinbauer I , Pauli H , Gottfried M , Brooker R , Nagy L , Theurillat J‐P , Holten JI , Abdaladze O , Benito J‐L *et al* 2007 Weak and variable relationships between environmental severity and small‐scale co‐occurrence in alpine plant communities. Journal of Ecology 95: 1284–1295.

[nph15290-bib-0021] Dullinger S , Willner W , Plutzar C , Englisch T , Schratt‐Ehrendorfer L , Moser D , Ertl S , Essl F , Niklfeld H . 2012b Post‐glacial migration lag restricts range filling of plants in the European Alps. Global Ecology and Biogeography 21: 829–840.

[nph15290-bib-0022] Dye DG . 2002 Variability and trends in the annual snow‐cover cycle in Northern Hemisphere land areas, 1972–2000. Hydrological Processes 16: 3065–3077.

[nph15290-bib-0023] Elmendorf SC , Henry GHR , Hollister RD , Bjork RG , Boulanger‐Lapointe N , Cooper EJ , Cornelissen JHC , Day TA , Dorrepaal E , Elumeeva TG *et al* 2012 Plot‐scale evidence of tundra vegetation change and links to recent summer warming. Nature Climate Change 2: 453–457.

[nph15290-bib-0024] Engler R , Randin C , Thuiller W , Dullinger S , Zimmermann NE , Araújo MB , Pearman PB , Le Lay G , Piédallu C , Albert CH *et al* 2011 21st century climate change threatens mountain flora unequally across Europe. Global Change Biology 17: 2330–2341.

[nph15290-bib-0025] Fournier D , Skaug H , Ancheta J , Ianelli J , Magnusson A , Maunder M , Nielsen A , Sibert J . 2012 AD Model Builder: using automatic differentiation for statistical inference of highly parameterized complex nonlinear models. Optimization Methods and Software 27: 233–249.

[nph15290-bib-0026] Friedel H . 1961 Schneedeckendauer und Vegetationsverteilungen im Gelände. Mitteilungen der Forstlichen Bundesversuchsanstalt Mariabrunn (Wien) 59: 317–369.

[nph15290-bib-0027] Giménez‐Benavides L , Escudero A , Iriondo JM . 2007 Reproductive limits of a late‐flowering high‐mountain Mediterranean plant along an elevational climate gradient. New Phytologist 173: 367–382. 17204083 10.1111/j.1469-8137.2006.01932.x

[nph15290-bib-0028] Gobiet A , Kotlarski S , Beniston M , Heinrich G , Rajczak J , Stoffel M . 2014 21st century climate change in the European Alps – a review. Science of the Total Environment 493: 1138–1151. 23953405 10.1016/j.scitotenv.2013.07.050

[nph15290-bib-0029] Gottfried M , Hantel M , Maurer C , Toechterle R , Pauli H , Grabherr G . 2011 Coincidence of the alpine‐nival ecotone with the summer snowline. Environmental Research Letters 6: 014013.

[nph15290-bib-0030] Gottfried M , Pauli H , Futschik A , Akhalkatsi M , Barancok P , Benito Alonso JL , Coldea G , Dick J , Erschbamer B , Fernandez Calzado MR *et al* 2012 Continent‐wide response of mountain vegetation to climate change. Nature Climate Change 2: 111–115.

[nph15290-bib-0031] Gottfried M , Pauli H , Grabherr G . 1998 Prediction of vegetation patterns at the limits of plant life: a new view of the alpine‐nival ecotone. Arctic and Alpine Research 30: 207–221.

[nph15290-bib-0032] Gottfried M , Pauli H , Reiter K , Grabherr G . 1999 A fine‐scaled predictive model for changes in species distribution patterns of high mountain plants induced by climate warming. Diversity and Distributions 5: 241–251.

[nph15290-bib-0033] Grabherr G . 1993 Caricetea curvulae In: Grabherr G, Mucina L, eds. Die Pflanzengesellschaften Österreichs ‐ Teil II. Jena, Germany: Gustav Fischer Verlag, 343–381.

[nph15290-bib-0034] Grabherr G , Gottfried M , Pauli H . 1994 Climate effects on mountain plants. Nature 369: 448. 10.1038/369448a0 23320303

[nph15290-bib-0035] Grabherr G , Gottfried M , Pauli H . 2001 Long‐term monitoring of mountain peaks in the Alps In: Burga CA, Kratochwil A, eds. Biomonitoring: general and applied aspects on regional and global scales. Dordrecht, the Netherlands: Tasks for Vegetation Science, Kluwer, 153–177.

[nph15290-bib-0036] Grabherr G , Gottfried M , Pauli H . 2010 Climate change impacts in alpine environments. Geography Compass 4: 1133–1153.

[nph15290-bib-0037] Grytnes J‐A , Kapfer J , Jurasinski G , Birks HH , Henriksen H , Klanderud K , Odland A , Ohlson M , Wipf S , Birks HJB . 2014 Identifying the driving factors behind observed elevational range shifts on European mountains. Global Ecology and Biogeography 23: 876–884.

[nph15290-bib-0038] Hagedorn F , Shiyatov SG , Mazepa VS , Devi NM , Grigor'ev AA , Bartysh AA , Fomin VV , Kapralov DS , Terent'ev M , Bugmann H *et al* 2014 Treeline advances along the Urals mountain range – driven by improved winter conditions? Global Change Biology 20: 3530–3543. 24756980 10.1111/gcb.12613

[nph15290-bib-0039] Hammer W , Ohnesorge T , Sander B , Kerner‐Marilaun F . 1929 Geologische Spezialkarte der Republik Österreich, 1:75.000, Blatt 5146, Ötzthal. Wien, Austria: Geologische Bundesanstalt.

[nph15290-bib-0040] Harsch MA , Hulme PE , McGlone MS , Duncan RP . 2009 Are treelines advancing? A global meta‐analysis of treeline response to climate warming. Ecology Letters 12: 1040–1049. 19682007 10.1111/j.1461-0248.2009.01355.x

[nph15290-bib-0041] Hartmann DL , Klein Tank AMG , Rusticucci M , Alexander LV , Bronnimann S , Charabi Y , Dentener FJ , Dlugokencky EJ , Easterling DR , Kaplan A *et al* 2013 Observations: atmosphere and surface In: Stocker TF, Qin D, Plattner G‐K, Tignor M, Allen SK, Boschung J, Nauels A, Xia Y, Bex V, Midgley PM, eds. Climate Change 2013: the physical science basis. Contribution of Working Group I to the Fifth Assessment Report of the Intergovernmental Panel on Climate Change. Cambridge, UK: Cambridge University Press.

[nph15290-bib-0042] Hervé M . 2016 RVAideMemoire: diverse basic statistical and graphical functions. R package version 0.9‐55. [WWW document] URL http://CRAN.R-project.org/package=RVAideMemoire [accessed 24 May 2018].

[nph15290-bib-0043] Hofmann K , Lamprecht A , Pauli H , Illmer P . 2016 Distribution of prokaryotic abundance and microbial nutrient cycling across a high‐alpine altitudinal gradient in the Austrian Alps is affected by vegetation, temperature, and soil nutrients. Soil Microbiology 72: 704–716. 10.1007/s00248-016-0803-z 27401822

[nph15290-bib-0044] Holzinger B , Hülber K , Camenisch M , Grabherr G . 2008 Changes in plant species richness over the last century in the eastern Swiss Alps: elevational gradient, bedrock effects and migration rates. Plant Ecology 195: 179–196.

[nph15290-bib-0045] Jiménez Cisneros BE , Oki T , Arnell NW , Benito G , Cogley JG , Döll P , Jiang T , Mwakalila SS . 2014 Freshwater resources In: Field CB, Barros VR, Dokken DJ, Mach KJ, Mastrandrea MD, Bilir TE, Chatterjee M, Ebi KL, Estrada YO, Genova RC *et al*, eds. Climate Change 2014: impacts, adaptation, and vulnerability. Part A: Global and sectoral aspects. Contribution of Working Group II to the Fifth Assessment Report of the Intergovernmental Panel on Climate Change. Cambridge, UK: Cambridge University Press, 229–269.

[nph15290-bib-0046] Kirkpatrick JB , Nunez M , Bridle KL , Parry J , Gibson N . 2017 Causes and consequences of variation in snow incidence on the high mountains of Tasmania, 1983–2013. Australian Journal of Botany 65: 214–224.

[nph15290-bib-0047] Klanderud K , Birks HJB . 2003 Recent increases in species richness and shifts in altitudinal distributions of Norwegian mountain plants. Holocene 13: 1–6.

[nph15290-bib-0048] Körner C . 2003 Alpine plant life: functional plant ecology of high mountain ecosystems. Berlin, Germany: Springer.

[nph15290-bib-0049] Körner C , Larcher W . 1988 Plant life in cold climates In: Long SF, Woodward FI, eds. Plant and temperature. Symp. Soc. Exp. Biol., 42. Cambridge, UK: The Company of Biologists, 25–57. 3270208

[nph15290-bib-0050] Kovats RS , Valentini R , Bouwer LM , Georgopoulou E , Jacob D , Martin E , Rounsevell M , Soussana J‐F . 2014 Europe In: Barros VR, Field CB, Dokken DJ, Mastrandrea MD, Mach KJ, Bilir TE, Chatterjee M, Ebi KL, Estrada YO, Genova RC *et al*, eds. Climate Change 2014: impacts, adaptation, and vulnerability. Part B: Regional aspects. Contribution of Working Group II to the Fifth Assessment Report of the Intergovernmental Panel on Climate Change. Cambridge, UK: Cambridge University Press, 1267–1326.

[nph15290-bib-0051] Kullman L . 2002 Rapid recent range‐margin rise of tree and shrub species in the Swedish Scandes. Journal of Ecology 90: 68–77.

[nph15290-bib-0052] Kuussaari M , Bommarco R , Heikkinen RK , Helm A , Krauss J , Lindborg R , Öckinger E , Pärtel M , Pino J , Rodà F *et al* 2009 Extinction debt: a challenge for biodiversity conservation. Trends in Ecology & Evolution 24: 564–571. 19665254 10.1016/j.tree.2009.04.011

[nph15290-bib-0053] Landolt E , Bäumler B , Erhardt A , Hegg O , Klötzli F , Lämmler W , Nobis M , Rudmann‐Maurer K , Schweingruber FH , Theurillat J‐P *et al* 2010 Flora indicativa: Ökologische Zeigerwerte und biologische Kennzeichen zur Flora der Schweiz und der Alpen/Ecological indicator values and biological attributes of the flora of Switzerland and the Alps. Bern, Switzerland: Haupt Verlag.

[nph15290-bib-0054] Larcher W , Wagner J , Lütz C . 1997 The effect of heat on photosynthesis, dark respiration and cellular ultrastructure of the arctic‐alpine psychrophyte *Ranunculus glacialis* . Photosynthetica 34: 219–232.

[nph15290-bib-0055] Larigauderie A , Körner C . 1995 Acclimation of leaf dark respiration to temperature in alpine and lowland plant species. Annals of Botany 76: 245–252.

[nph15290-bib-0056] Lenoir J , Svenning J‐C . 2013 Latitudinal and elevational range shifts under contemporary climate change In: Levin SA ed. Encyclopedia of biodiversity, *2^nd^ edn* Oxford, UK: Academic Press, 599–611.

[nph15290-bib-0057] Lenth RV . 2016 Least‐squares means: the R package lsmeans. Journal of Statistical Software 69: 1–33.

[nph15290-bib-0058] Lesica P . 2014 Arctic‐alpine plants decline over two decades in Glacier National Park, Montana, U.S.A. Arctic Antarctic and Alpine Research 46: 327–332.

[nph15290-bib-0059] Luterbacher J , Naish T , Osborn T , Otto‐Bliesner B , Quinn T , Ramesh R , Rojas M , Shao X , Timmermann A 2013 Information from Paleoclimate Archives In: Stocker TF, Qin D, Plattner G‐K, Tignor M, Allen SK, Boschung J, Nauels A, Xia Y, Bex V, Midgley PM, eds. Climate Change 2013: the physical science basis. Contribution of Working Group I to the Fifth Assessment Report of the Intergovernmental Panel on Climate Change. Cambridge, UK: Cambridge University Press.

[nph15290-bib-0060] Malanson GP , Rose JP , Schroeder PJ , Fagre DB . 2011 Contexts for change in alpine tundra. Physical Geography 32: 97–113.

[nph15290-bib-0061] Marty C , Meister R . 2012 Long‐term snow and weather observations at Weissfluhjoch and its relation to other high‐altitude observatories in the Alps. Theoretical and Applied Climatology 110: 573–583.

[nph15290-bib-0062] Matteodo M , Wipf S , Stöckli V , Rixen C , Vittoz P . 2013 Elevation gradient of successful plant traits for colonizing alpine summits under climate change. Environmental Research Letters 8: 024043.

[nph15290-bib-0063] Mirtl M , Bahn M , Battin T , Borsdorf A , Dirnböck T , Englisch M , Erschbamer B , Fuchsberger J , Gaube V , Grabherr G *et al* 2015 Forschung für die Zukunft – LTER‐Austria White Paper 2015 zur Lage und Ausrichtung von prozessorientierter Ökosystemforschung, Biodiversitäts‐ und Naturschutzforschung sowie sozio‐ökologischer Forschung in Österreich. Wien, Austria: LTER‐Austria Schriftenreihe, Vol. 2.

[nph15290-bib-0064] Mountain Research Initiative EDW Working Group . 2015 Elevation‐dependent warming in mountain regions of the world. Nature Climate Change 5: 424–430.

[nph15290-bib-0065] Odland A , Høitomt T , Olsen SL . 2010 Increasing vascular plant richness on 13 high mountain summits in Southern Norway since the early 1970s. Arctic Antarctic and Alpine Research 42: 458–470.

[nph15290-bib-0066] Ohmura A . 2012 Enhanced temperature variability in high‐altitude climate change. Theoretical and Applied Climatology 110: 499–508.

[nph15290-bib-0067] Oksanen J , Blanchet FG , Friendly M , Kindt R , Legendre P , McGlinn D , Minchin PR , O'Hara RB , Simpson GL , Solymos P *et al* 2018 vegan: Community Ecology Package. R package version 2.4‐6. [WWW document] URL https://CRAN.R-project.org/package=vegan [accessed 24 May 2018].

[nph15290-bib-0068] Opedal OH , Armbruster WS , Graae BJ . 2015 Linking small‐scale topography with microclimate, plant species diversity and intra‐specific trait variation in an alpine landscape. Plant Ecology & Diversity 8: 305–315.

[nph15290-bib-0069] Pauli H , Gottfried M , Dullinger S , Abdaladze O , Akhalkatsi M , Benito Alonso JL , Coldea G , Dick J , Erschbamer B , Fernández Calzado R *et al* 2012 Recent plant diversity changes on Europe's mountain summits. Science 336: 353–355. 22517860 10.1126/science.1219033

[nph15290-bib-0070] Pauli H , Gottfried M , Grabherr G . 1999 Vascular plant distribution patterns at the low‐temperature limits of plant life – the alpine‐nival ecotone of Mount Schrankogel (Tyrol, Austria). Phytocoenologia 29: 297–325.

[nph15290-bib-0071] Pauli H , Gottfried M , Lamprecht A , Niessner S , Rumpf S , Winkler M , Steinbauer K , Grabherr G . 2015 The GLORIA field manual – standard Multi‐Summit approach, supplementary methods and extra approaches. Vienna, Austria: GLORIA‐Coordination, Austrian Academy of Sciences & University of Natural Resources and Life Sciences.

[nph15290-bib-0072] Pauli H , Gottfried M , Reiter K , Klettner C , Grabherr G . 2007 Signals of range expansions and contractions of vascular plants in the high Alps: observations (1994–2004) at the GLORIA master site Schrankogel, Tyrol, Austria. Global Change Biology 13: 147–156.

[nph15290-bib-0073] Purtscheller F . 1978 Ö tztaler und Stubaier Alpen. ‐ Sammlung geol. Führer, 53, 2. verb. Auflage, 128 S., 1 geol. Kt., 21 Abb. Berlin, Germany: Borntraeger.

[nph15290-bib-0074] R Core Team . 2015 R: a language and environment for statistical computing. Vienna, Austria: R Foundation for Statistical Computing [WWW document] URL http://www.R-project.org [accessed 24 May 2018].

[nph15290-bib-0075] Rumpf SB , Hülber K , Klonner G , Moser D , Schutz M , Wessely J , Willner W , Zimmermann NE , Dullinger S . 2018 Range dynamics of mountain plants decrease with elevation. Proceedings of the National Academy of Sciences, USA 115: 1848–1853. 10.1073/pnas.1713936115 PMC5828587 29378939

[nph15290-bib-0076] Scherrer D , Körner C . 2011 Topographically controlled thermal‐habitat differentiation buffers alpine plant diversity against climate warming. Journal of Biogeography 38: 406–416.

[nph15290-bib-0077] Schmidli J , Goodess CM , Frei C , Haylock MR , Hundecha Y , Ribalaygua J , Schmith T . 2007 Statistical and dynamical downscaling of precipitation: an evaluation and comparison of scenarios for the European Alps. Journal of Geophysical Research. Atmospheres 112(D4): D04105.

[nph15290-bib-0078] Steger C , Kotlarski S , Jonas T , Schar C . 2013 Alpine snow cover in a changing climate: a regional climate model perspective. Climate Dynamics 41: 735–754.

[nph15290-bib-0079] Steinbauer MJ , Grytnes JA , Jurasinski G , Kulonen A , Lenoir J , Pauli H , Rixen C , Winkler M , Bardy‐Durchhalter M , Barni E *et al* 2018 Accelerated increase in plant species richness on mountain summits is linked to warming. Nature 556: 231–234. 29618821 10.1038/s41586-018-0005-6

[nph15290-bib-0080] Stöckli V , Wipf S , Nilsson C , Rixen C . 2011 Using historical plant surveys to track biodiversity on mountain summits. Plant Ecology & Diversity 4: 415–425.

[nph15290-bib-0081] Theurillat J‐P , Guisan A . 2001 Potential impact of climate change on vegetation in the European Alps: a review. Climatic Change 50: 77–109.

[nph15290-bib-0082] UNEP‐WCMC & IUCN . 2018 Protected planet: The world database on protected areas (WDPA)/The global database on protected areas management effectiveness (GD‐PAME). [WWW document] URL https://www.protectedplanet.net/. [accessed 11 May 2018].

[nph15290-bib-0083] Vanneste T , Michelsen O , Graae BJ , Kyrkjeeide MO , Holien H , Hassel K , Lindmo S , Kapas RE , De Frenne P . 2017 Impact of climate change on alpine vegetation of mountain summits in Norway. Ecological Research 32: 579–593.

[nph15290-bib-0084] Venables WN , Ripley BD . 2002 Modern applied statistics with S. New York, NY, USA: Springer.

[nph15290-bib-0085] Vittoz P , Bodin J , Ungricht S , Burga CA , Walther G‐R . 2008 One century of vegetation change on Isla Persa, a nunatak in the Bernina massif in the Swiss Alps. Journal of Vegetation Science 19: 671–680.

[nph15290-bib-0086] Walther G‐R , Beißner S , Burga CA . 2005 Trends in the upward shift of alpine plants. Journal of Vegetation Science 16: 541–548.

[nph15290-bib-0087] Wang Q , Fan X , Wang M . 2016 Evidence of high‐elevation amplification versus Arctic amplification. Scientific Reports 6: 19219. 26753547 10.1038/srep19219 PMC4709741

[nph15290-bib-0088] Wickham H . 2009 ggplot2: elegant graphics for data analysis. New York, NY, USA: Springer.

[nph15290-bib-0089] Wipf S , Stöckli V , Herz K , Rixen C . 2013 The oldest monitoring site of the Alps revisited: accelerated increase in plant species richness on Piz Linard summit since 1835. Plant Ecology and Diversity 6: 447–455.

[nph15290-bib-0090] de Witte LC , Stöcklin J . 2010 Longevity of clonal plants: why it matters and how to measure it. Annals of Botany 106: 859–870. 20880935 10.1093/aob/mcq191 PMC2990663

