# Supplementary material for: Climate change leads to accelerated transformation of high‐elevation vegetation in the central Alps
Source: New Phytol. 2018 Jun 25;220(2):447–59. doi: 10.1111/nph.15290 (PMC6175417; doi:10.1111/nph.15290)
Supplement: Supplementary file 1 — Fig. S1 Location of permanent plots on Mount Schrankogel with transect details. Fig. S2 Correlation between thermic indicator and temperature sum on Mount Schrankogel. Fig. S3 Vascular plant species richness per altitudinal rank in the survey years 1994, 2004 and 2014. Fig. S4 Relative colonisation and disappearance per altitudinal rank in the periods 1994–2004 and 2004–2014. Fig. S5 Cover sum of vascular plant species per altitudinal rank in the survey years 1994, 2004 and 2014. Fig. S6 Correlation between the changes in thermic indicator and changes in soil moisture indicator per plot in the periods 1994–2004 and 2004–2014. Fig. S7 Topographic parameters per block on Mount Schrankogel. Fig. S8 Non‐metric multidimensional scaling (NMDS) of topographic parameters of plots. Table S1 Setup of permanent plots for the monitoring of vascular plant species on Mount Schrankogel Table S2 Frequency of annual plant species Table S3 Vascular plant species per altitudinal rank in the survey years 1994, 2004 and 2014 Table S4 Vascular plant species richness in the survey years 1994, 2004 and 2014 Table S5 Changes in vascular plant species richness in the periods 1994–2004, 1994–2014 and 2004–2014 Table S6 Vascular plant species richness per altitudinal rank in the survey years 1994, 2004 and 2014 Table S7 Changes in vascular plant species richness per altitudinal rank in the periods 1994–2004, 1994–2014 and 2004–2014 Table S8 Number of colonising and disappearing species at the end of periods 1994–2004 and 2004–2014 Table S9 Differences between numbers of colonising and disappearing species within and among the periods 1994–2004 and 2004–2014 Table S10 Relative colonisation and disappearance per altitudinal rank in the periods 1994–2004 and 2004–2014 Table S11 Changes in relative colonisation and disappearance per altitudinal rank in the periods 1994–2004 and 2004–2014 Table S12 Cover sum of species in the survey years 1994, 2004 and 2014 Table S13 Changes in cover sum of specie [file NPH-220-447-s001.pdf]

New Phytologist Supporting Information

Climate change leads to accelerated transformation of high-elevation vegetation in the central Alps

Andrea Lamprecht, Philipp Robert Semenchuk, Klaus Steinbauer, Manuela Winkler and Harald Pauli

Article acceptance date: 23 May 2018

The following Supporting Information is available for this article:

Fig. S1 Location of permanent plots on Mount Schrankogel with transect details 3
Fig. S2 Correlation between thermic indicator and temperature sum on Mount Schrankogel 5
Fig. S3 Vascular plant species richness per altitudinal rank in the survey years 1994, 2004 and 2014 7
Fig. S4 Relative colonisation and disappearance per altitudinal rank in the periods 1994–2004 and 2004-2014 10
Fig. S5 Cover sum of vascular plant species per altitudinal rank in the survey years 1994, 2004 and 2014 12
Fig. S6 Correlation between the changes of thermic indicator and changes of soil moisture indicator per plot in the periods 1994-2004 and 2004-2014 15
Fig. S7 Topographic parameters per block on Mount Schrankogel 16
Fig. S8 Non-metric multidimensional scaling (NMDS) of topographic parameters of plots 17

Table S1 Setup of permanent plots for monitoring of vascular plant species on Mount Schrankogel. 4
Table S2 Frequency of annual plant species. 4
Table S3 Vascular plant species per altitudinal rank in the survey years 1994, 2004 and 2014 5
Table S4 Vascular plant species richness in the survey years 1994, 2004 and 2014 6
Table S5 Changes in vascular plant species richness in the periods 1994-2004, 1994-2014 and 2004-2014 6
Table S6 Vascular plant species richness per altitudinal rank in the survey years 1994, 2004 and 2014 7
Table S7 Changes in vascular plant species richness per altitudinal rank in the periods 1994-2004, 1994-2014 and 2004-2014 8
Table S8 Number of colonising and disappearing species at the end of the periods 1994-2004 and 2004-2014 8
Table S9 Differences between numbers of colonising and disappearing species within and among the periods 1994-2004 and 2004-2014 9
Table S10 Relative colonisation and disappearance per altitudinal rank in the periods 1994-2004 and 2004-2014 ..10

Table S11 Changes in relative colonisation and disappearance per altitudinal rank in the periods 1994-2004 and 2004-2014 11
Table S12 Cover sum of vascular plant species in the survey years 1994, 2004 and 2014 11
Table S13 Changes in cover sum of species in the periods 1994-2004, 1994-2014 and 2004-2014 11
Table S14 Cover sum of vascular plant species per altitudinal rank in the survey years 1994, 2004 and 2014 12
Table S15 Changes in mean cover sum of vascular plant species per altitudinal rank in the periods 1994-2004, 1994-2014 and 2004-2014 13
Table S16 Thermic indicator in the survey years 1994, 2004 and 2014 13
Table S17 Thermophilisation in the periods 1994-2004, 1994-2014 and 2004-2014 14
Table S18 Changes in thermophilisation between the periods 1994-2004 and 2004-2014 14
Table S19 Soil moisture indicator in the survey years 1994, 2004 and 2014 14
Table S20 Change in soil moisture indicator in the periods 1994-2004, 1994-2014 and 2004-2014 15
Table S21 Changes in Δ soil moisture indicator between the periods 1994-2004 and 2004-2014 15
Table S22 Differences among blocks on Mount Schrankogel 17
Table S23 Vascular plant species richness in the survey years 1994 and 2014 18
Table S24 Changes in vascular plant species richness in the period 1994-2014 18
Table S25 Number of colonising and disappearing species in the period 1994-2014 18
Table S26 Differences between numbers of colonising and disappearing species in the period 1994-2014 19
Table S27 Thermic indicator in the survey years 1994 and 2014 19
Table S28 Thermophilisation in the period 1994-2014 19
Table S29 Soil moisture indicator in the survey years 1994 and 2014 20
Table S30 Changes in soil moisture in the period 1994-2014 20

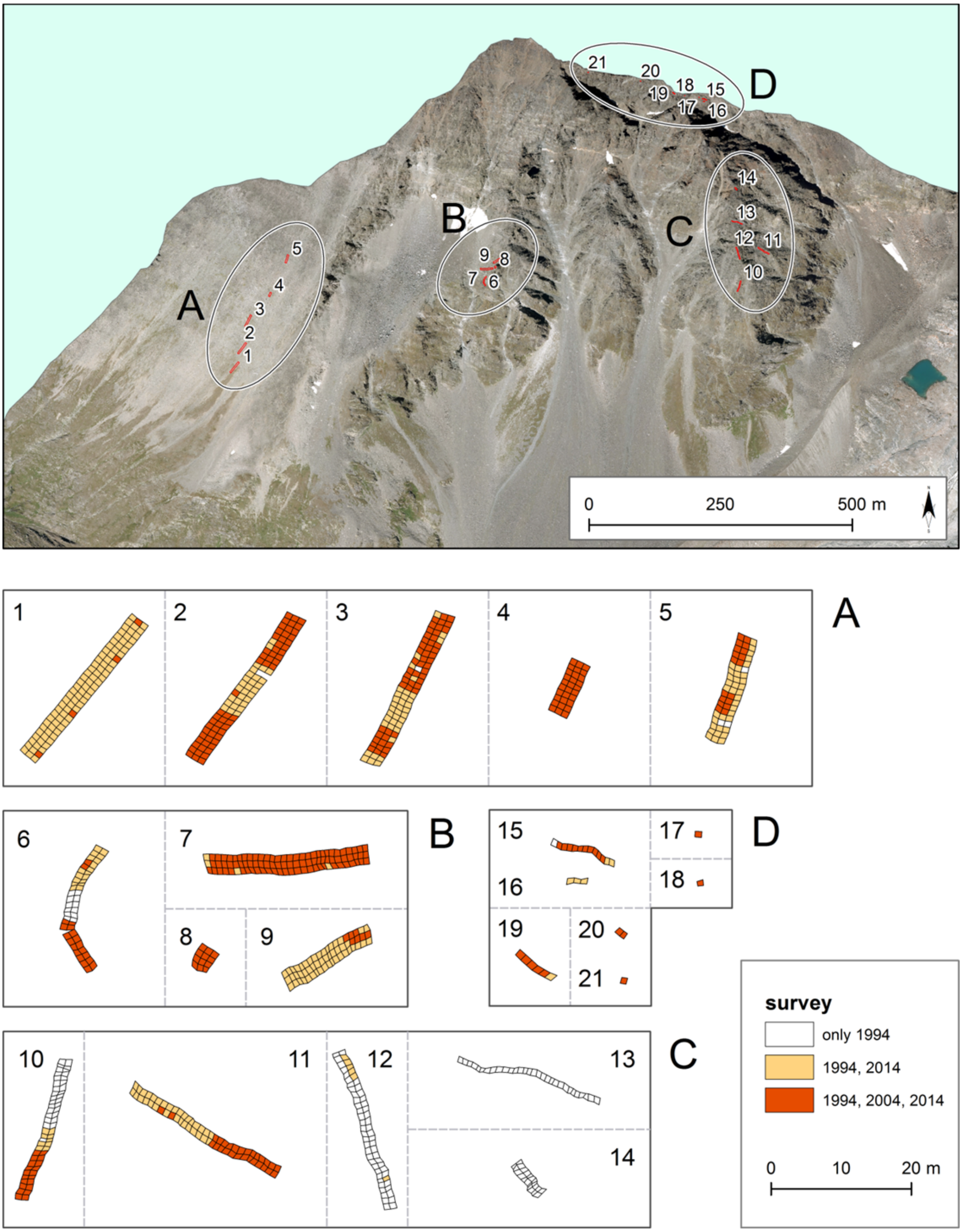

Fig. S1 *Location of permanent plots on Mount Schrankogel with transect details. Plots are grouped in transects clustered in four topographic blocks: A) uniformly shaped SW- slope, rich in scree; B) rugged S-facing ridge, C) rugged S-SE facing ridge and D) S-facing high-elevation plots along the E-ridge. Orthophotos (© Land Tirol) modified with ArcGIS 10.3. for Desktop, Esri Inc. Inserts show a detailed view of each transect.*

Table S1 *Setup of permanent plots for monitoring of vascular plant species on Mount Schrankogel. Within four blocks (A, B, C, D), 21 transects are situated, each consisting of 1 up to 90 1-m² permanent plots. The number of plots surveyed in all three survey-years 1994, 2004 and 2014 and the number of plots of an additional dataset including only plots from 1994 and 2014 (data only given in supplementary; Tables S23 – S30), as well as the altitudinal range of each transect are given. Transects 12-14 were not resurveyed.*

Block	Transect	Number of plots	Number of plots of additional dataset (1994 – 2014)	Altitude [m]
A	1	4	90	2911-2927
A	2	59	88	2936-2952
A	3	52	89	2970-2987
A	4	30	30	3022-3028
A	5	18	51	3074-3084
	sum A	163	348	2911-3084
B	6	20	38	3072-3086
B	7	85	89	3088-3105
B	8	12	12	3108-3109
B	9	7	56	3109-3120
	sum B	124	195	3072-3120
C	10	20	29	3084-3094
C	11	28	56	3100-3115
	sum C	48	85	3084-3115
D	15	10	12	3348-3354
D	16	0	3	3348-3349
D	17	1	1	3360
D	18	1	1	3382
D	19	5	6	3392-3349
D	20	2	2	3418-3420
D	21	1	1	3457
	sum D	20	26	3348-3457
	total	355	654	2911-3457

Table S2 *Frequency of annual plant species. Number of plots where annual species occurred in the survey years 1994, 2004 and 2014 in the permanent plots on Mount Schrankogel.*

Year	*Euphrasia minima*	*Gentianella tenella*
1994	37	0
2004	14	0
2014	166	6

Table S3 *Vascular plant species per altitudinal rank in the survey years 1994, 2004 and 2014 over the whole study area on Mount Schrankogel. The altitudinal rank (AR) denotes a species' distribution centre along the elevation gradient (range; after Gottfried et al. 2012). Shown are the total number of species over the whole study area in all three study years, the number of species over the whole study area present in 1994, 2004 and 2014, respectively, and all vascular plant species per altitudinal rank. Species present in all three surveys are given in bold. Species which occure for the first time in the whole study area in a survey year are marked with *, which disappeared completely in one survey year with †, in each case with the corresponding year.*

Altitudinal rank	Range	Number of species All	1994	2004	2014	Species
AR1	Subnival – nival	6	6	6	6	***Androsace alpina; Cerastium uniflorum; Poa laxa; Ranunculus glacialis; Saxifraga bryoides; Saxifraga oppositifolia*** **subsp.** ***oppositifolia***
AR2	Alpine – subnival	20	20	20	20	***Arenaria ciliata*** **subsp.** ***ciliata; Cardamine resedifolia; Draba fladnizensis; Erigeron uniflorus; Festuca intercedens; Gentiana bavarica; Geum reptans; Leucanthemopsis alpina*** **subsp.** ***alpina; Linaria alpina; Luzula spicata; Oreochloa disticha; Pedicularis aspleniifolia; Potentilla frigida; Primula glutinosa; Saxifraga androsacea; Saxifraga exarata*** **subsp.** ***exarata; Saxifraga seguieri; Silene acaulis*** **subsp.** ***bryoides; Trisetum spicatum*** **subsp.** ***spicatum; Veronica alpina***
AR3	Alpine	21	18	19	20	*Agrostis alpina*[*14]***; Agrostis rupestris; Antennaria carpatica; Carex curvula*** **subsp.** ***curvula; Cerastium cerastoides;*** *Cerastium pedunculatum*[*14]***; Gentiana brachyphylla*** **subsp.** ***brachyphylla; Juncus jacquinii; Minuartia sedoides; Minuartia verna*** **subsp.** ***verna; Omalotheca supina;*** *Oxyria digyna*[*04, †14]***; Phyteuma hemisphaericum; Poa alpina; Primula minima; Sagina saginoides*** **subsp.** ***saginoides; Salix herbacea; Sedum alpestre; Senecio carniolicus; Sibbaldia procumbens; Veronica bellidioides***
AR4	(Montane -) treeline – alpine	14	6	8	13	*Alchemilla vulgaris* agg.[*14]; *Anthoxanthum odoratum* subsp. *alpinum*[*04]***; Avenula versicolor*** **subsp.** ***versicolor;*** *Botrychium lunaria*[*04]***; Festuca halleri*** **subsp.** ***halleri; Geum montanum;*** *Homogyne alpina*[*14]***; Leontodon pyrenaicus*** **subsp.** ***helveticus; Polygonum viviparum;*** *Potentilla aurea* subsp. *aurea*[*14]; *Potentilla crantzii*[*14]; *Primula hirsuta*[*14]; *Ranunculus grenieranus*[†04]; *Taraxacum apenninum* agg.[*04]

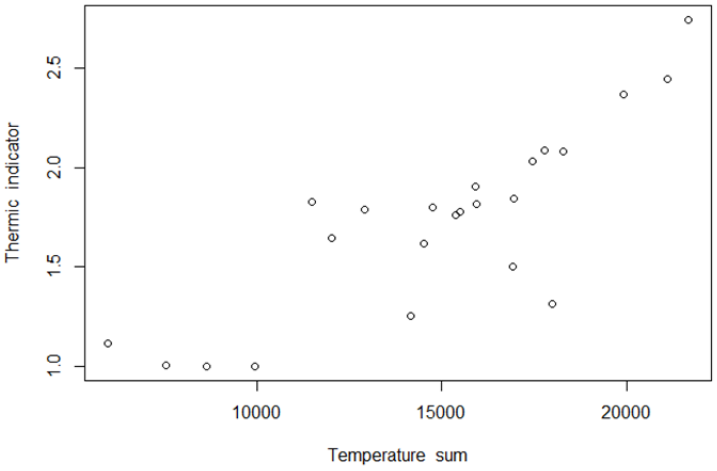

Fig. S2 *Correlation between thermic indicator and temperature sum on Mount Schrankogel. The thermic indicator per plot was calculated as the mean altitudinal rank (1 = subnival-nival, 2 = alpine-subnival, 3 = alpine, 4 = treeline-alpine species) weighted with the respective species' cover (Gottfried et al., 2012). The shown thermic indicator values are means from five plots in the vicinity of each temperature logger. Temperature sums represent annual mean values of hourly measurements from soil temperature loggers (n=23) summed up above a threshold of 3 °C in the period 01.08.2012 – 31.07.2014. The correlation was highly significant (linear mixed effect model; $p < 0.0001$; df = 21; marginal $R^2 = 0.66$, conditional $R^2 = 0.7$: calculated using the function r.squaredGLMM from the R-package MuMIn; Burnham & Anderson, 2002).*

Vascular plant species richness

Table S4 *Vascular plant species richness in the survey years 1994, 2004 and 2014 on Mount Schrankogel.* ***a)*** *Mean and standard error (SE) of raw data and* ***b)*** *least-square mean (lsmean), standard error (SE), degrees of freedom (df) and 95% confidence interval (LCL and UCL), derived from generalized linear mixed effect models with a penalized quasi-likelihood estimation and a Poisson distribution, of the species richness per plot in the three survey years For plot numbers per block see Table S1.*

		(a) Raw data		(b) Model				
Year	Block	mean	SE	lsmean	SE	df	LCL	UCL
1994	All	10.84	0.21	8.81	1.26	3	5.59	13.90
2004	All	12.61	0.23	10.26	1.47	3	6.50	16.17
2014	All	13.08	0.22	10.64	1.52	3	6.74	16.77
1994	A	11.46	0.30	10.07	1.26	4	7.11	14.26
2004	A	14.15	0.32	12.43	1.56	4	8.78	17.60
2014	A	14.53	0.29	12.77	1.60	4	9.02	18.08
1994	B	9.75	0.31	8.75	1.15	3	5.76	13.28
2004	B	10.60	0.33	9.51	1.25	3	6.26	14.43
2014	B	11.23	0.31	10.07	1.32	3	6.63	15.29
1994	C	13.65	0.51	13.32	0.47	1	8.47	20.94
2004	C	15.13	0.52	14.76	0.52	1	9.42	23.13
2014	C	15.54	0.58	15.17	0.54	1	9.69	23.75
1994	D	5.80	0.46	5.54	0.45	19	4.68	6.56
2004	D	6.60	0.49	6.31	0.50	19	5.34	7.45
2014	D	6.85	0.53	6.55	0.52	19	5.55	7.73

Table S5 *Changes in vascular plant species richness in the periods 1994-2004, 1994-2014 and 2004-2014 on Mount Schrankogel. Effect sizes (estimate), 95% confidence interval (LCL and UCL) and p-values of the species richness per plot between the initial survey 1994 and the resurveys 2004 and 2014, derived from generalized linear mixed effect models with a penalized quasi-likelihood estimation and a Poisson distribution. For plot numbers per block see Table S1.*

Block	Hypothesis	Estimate	LCL	UCL	p-value
All	2004 - 1994 == 0	0.15	0.13	0.18	<0.001
All	2014 - 1994 == 0	0.19	0.16	0.21	<0.001
All	2014 - 2004 == 0	0.04	0.01	0.06	<0.001
A	2004 - 1994 == 0	0.21	0.17	0.25	<0.001
A	2014 - 1994 == 0	0.24	0.20	0.27	<0.001
A	2014 - 2004 == 0	0.03	-0.01	0.06	0.152
B	2004 - 1994 == 0	0.08	0.04	0.12	<0.001
B	2014 - 1994 == 0	0.14	0.10	0.18	<0.001
B	2014 - 2004 == 0	0.06	0.02	0.10	0.001
C	2004 - 1994 == 0	0.10	0.06	0.15	<0.001
C	2014 - 1994 == 0	0.13	0.09	0.17	<0.001
C	2014 - 2004 == 0	0.03	-0.02	0.07	0.300
D	2004 - 1994 == 0	0.13	0.02	0.24	0.014
D	2014 - 1994 == 0	0.17	0.06	0.27	0.001
D	2014 - 2004 == 0	0.04	-0.07	0.14	0.676

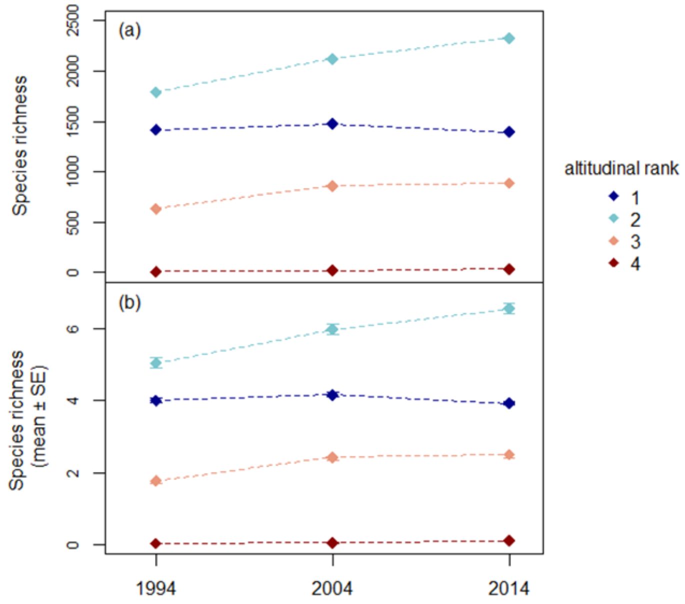

Fig. S3 *Vascular plant species richness per altitudinal rank in the survey years 1994, 2004 and 2014 on Mount Schrankogel. a) Sum of species counts over all 355 plots and b) mean and standard error of raw data of species richness per plot in each altitudinal rank. 1994-2004 and 1994-2014 were significantly different within altitudinal rank 2 and 3, respectively (generalized mixed effects models using penalized quasi likelihood and a negative binomial distribution; Tables S6, S7). For species numbers per altitudinal rank and year see Table S3.*

Table S6 *Vascular plant species richness per altitudinal rank (AR) in the survey years 1994, 2004 and 2014 on Mount Schrankogel.* ***a)*** *Sum of species counts over all plots, mean and standard error (SE) of raw data and* **b)** *least-square mean (lsmean), standard error (SE), degrees of freedom (df) and 95% confidence interval (LCL and UCL), derived from generalized linear mixed effect models with a penalized quasi-likelihood estimation and a negative binomial distribution, of the species richness per plot and altitudinal rank in the three survey years. For species numbers per altitudinal rank and year see Table S3.*

		(a) Raw data			(b) Model				
Year	AR	sum	mean	SE	lsmean	SE	df	LCL	UCL
1994	1	1418	3.99	0.06	3.65	0.32	3	2.77	4.81
2004	1	1478	4.16	0.06	3.82	0.33	3	2.90	5.05
2014	1	1394	3.93	0.06	3.62	0.32	3	2.74	4.77
1994	2	1790	5.04	0.14	4.62	0.40	3	3.50	6.09
2004	2	2122	5.98	0.14	5.36	0.47	3	4.06	7.06
2014	2	2329	6.56	0.14	5.83	0.51	3	4.42	7.69
1994	3	630	1.77	0.08	1.83	0.16	3	1.38	2.43
2004	3	860	2.42	0.10	2.33	0.20	3	1.76	3.08
2014	3	887	2.50	0.10	2.39	0.21	3	1.81	3.16
1994	4	10	0.03	0.01	1.09	0.20	3	0.60	1.98
2004	4	18	0.05	0.02	1.05	0.16	3	0.66	1.69
2014	4	34	0.10	0.02	1.14	0.14	3	0.77	1.69

Table S7 *Changes in vascular plant species richness per altitudinal rank (AR) in the periods 1994-2004, 1994-2014 and 2004-2014 on Mount Schrankogel. Effect sizes (estimate), 95% confidence interval (LCL and UCL) and p-values of the species richness per plot between the initial survey 1994 and the resurveys 2004 and 2014, derived from generalized linear mixed effect models with a penalized quasi-likelihood estimation and a negative binomial distribution. For species numbers per altitudinal rank and year see Table S3.*

AR	Hypothesis	Estimate	LCL	UCL	p-value
1	2004 - 1994 == 0	1.05	1.02	1.08	0.863
1	2014 - 1994 == 0	1.01	0.98	1.04	1.000
1	2014 - 2004 == 0	1.06	1.03	1.09	0.684
2	2004 - 1994 == 0	1.16	1.13	1.19	<.0001
2	2014 - 1994 == 0	1.26	1.23	1.30	<.0001
2	2014 - 2004 == 0	1.09	1.06	1.12	0.070
3	2004 - 1994 == 0	1.27	1.23	1.31	<.0001
3	2014 - 1994 == 0	1.30	1.26	1.35	<.0001
3	2014 - 2004 == 0	1.03	0.99	1.06	1.000
4	2004 - 1994 == 0	1.04	0.85	1.24	1.000
4	2014 - 1994 == 0	1.04	0.83	1.25	1.000
4	2014 - 2004 == 0	1.08	0.92	1.24	1.000

Colonisations and disappearances

Table S8 *Number of colonising and disappearing vascular plant species at the end of the periods 1994-2004 and 2004-2014 on Mount Schrankogel.* ***a)*** *Mean and standard error (SE) of raw data and* ***b)*** *least-square mean (lsmean), standard error (SE) and asymptotic confidence interval (LCL and UCL), derived from negative binomial generalized linear mixed-effects models, of numbers of colonising and disappearing species per plot between the initial survey 1994 and the resurveys 2004 and 2014. For plot numbers per block see Table S1.*

			(a) Raw data		(b) Model			
Type	Year	Block	mean	SE	lsmean	SE	LCL	UCL
Colonisations	2004	All	2.08	0.09	1.58	0.31	1.07	2.33
Disappearances	2004	All	0.31	0.03	0.23	0.05	0.15	0.36
Colonisations	2014	All	1.76	0.08	1.34	0.27	0.91	1.99
Disappearances	2014	All	1.29	0.07	0.98	0.20	0.66	1.45
Colonisations	2004	A	2.94	0.14	2.90	0.17	2.58	3.27
Disappearances	2004	A	0.25	0.04	0.25	0.04	0.18	0.34
Colonisations	2014	A	2.12	0.12	2.09	0.14	1.83	2.38
Disappearances	2014	A	1.73	0.12	1.71	0.12	1.49	1.96
Colonisations	2004	B	1.22	0.10	1.06	0.21	0.71	1.57
Disappearances	2004	B	0.37	0.05	0.32	0.08	0.20	0.51
Colonisations	2014	B	1.53	0.13	1.33	0.26	0.90	1.97
Disappearances	2014	B	0.90	0.10	0.79	0.16	0.52	1.18
Colonisations	2004	C	1.88	0.22	1.84	0.21	1.48	2.29
Disappearances	2004	C	0.40	0.09	0.39	0.09	0.25	0.61
Colonisations	2014	C	1.52	0.18	1.49	0.18	1.17	1.90
Disappearances	2014	C	1.10	0.18	1.08	0.15	0.82	1.43
Colonisations	2004	D	0.95	0.25	0.95	0.22	0.61	1.49
Disappearances	2004	D	0.15	0.11	0.15	0.09	0.05	0.47
Colonisations	2014	D	0.80	0.17	0.80	0.20	0.49	1.31
Disappearances	2014	D	0.55	0.15	0.55	0.17	0.30	0.99

Table S9 *Differences between numbers of colonising and disappearing vascular plant species within and among the periods 1994-2004 and 2004-2014 on Mount Schrankogel. Effect sizes (estimate), 95% confidence interval (LCL and UCL) and p-values of differences of numbers of colonising and disappearing species per plot over the entire study area and for each block between 1994, 2004 and 2014, derived from negative binomial generalized linear mixed-effects models with an ANOVA likelihood ratio test. For plot numbers per block see Table S1.*

Block	Hypothesis	Estimate	LCL	UCL	p-value
All	colonisations 2004 - colonisations 2014 == 0	0.16	0.01	0.31	0.03
All	disappearances 2004 - disappearances 2014 == 0	-1.43	-1.71	-1.15	0.001
All	disappearances 2004 - colonisations 2004 == 0	-1.91	-2.17	-1.64	0.001
All	disappearances 2014 - colonisations 2014 == 0	-0.32	-0.48	-0.15	0.001
All	colonisations 2004 - disappearances 2014 == 0	0.48	0.31	0.64	0.001
All	disappearances 2004 - colonisations 2014 == 0	-1.75	-2.02	-1.48	0.001
A	colonisations 2004 - colonisations 2014 == 0	0.33	0.15	0.51	0.001
A	disappearances 2004 - disappearances 2014 == 0	-1.93	-2.35	-1.51	0.001
A	disappearances 2004 - colonisations 2004 == 0	-2.46	-2.87	-2.05	0.001
A	disappearances 2014 - colonisations 2014 == 0	-0.2	-0.4	0	0.052
A	colonisations 2004 - disappearances 2014 == 0	0.53	0.34	0.72	0.001
A	disappearances 2004 - colonisations 2014 == 0	-2.13	-2.55	-1.71	0.001
B	colonisations 2004 - colonisations 2014 == 0	-0.23	-0.5	0.05	0.138
B	disappearances 2004 - disappearances 2014 == 0	-0.89	-1.33	-0.45	0.001
B	disappearances 2004 - colonisations 2004 == 0	-1.19	-1.61	-0.76	0.001
B	disappearances 2014 - colonisations 2014 == 0	-0.53	-0.83	-0.23	0.001
B	colonisations 2004 - disappearances 2014 == 0	0.3	-0.02	0.61	0.07
B	disappearances 2004 - colonisations 2014 == 0	-1.42	-1.83	-1	0.001
C	colonisations 2004 - colonisations 2014 == 0	0.21	-0.19	0.61	0.534
C	disappearances 2004 - disappearances 2014 == 0	-1.03	-1.71	-0.34	0.001
C	disappearances 2004 - colonisations 2004 == 0	-1.56	-2.2	-0.91	0.001
C	disappearances 2014 - colonisations 2014 == 0	-0.32	-0.78	0.14	0.276
C	colonisations 2004 - disappearances 2014 == 0	0.53	0.09	0.97	0.011
C	disappearances 2004 - colonisations 2014 == 0	-1.35	-2	-0.69	0.001
D	colonisations 2004 - colonisations 2014 == 0	0.17	-0.69	1.04	0.956
D	disappearances 2004 - disappearances 2014 == 0	-1.3	-2.96	0.36	0.181
D	disappearances 2004 - colonisations 2004 == 0	-1.85	-3.43	-0.26	0.015
D	disappearances 2014 - colonisations 2014 == 0	-0.37	-1.37	0.62	0.765
D	colonisations 2004 - disappearances 2014 == 0	0.55	-0.42	1.51	0.46
D	disappearances 2004 - colonisations 2014 == 0	-1.67	-3.28	-0.07	0.036

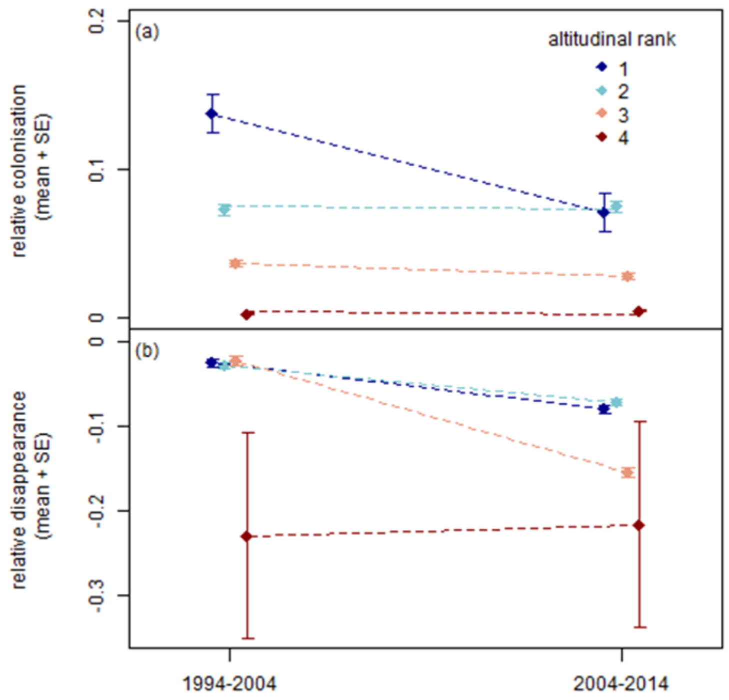

Fig. S4 *Relative colonisation and disappearance per altitudinal rank (AR) in the periods 1994–2004 and 2004-2014 on Mount Schrankogel. Shown are mean and standard error (SE) per species in each AR for a) relative colonisations and b) relative disappearances (Table S10). Relative colonisations of AR1 were significantly different between 1994-2004 and 2004-2014. Relative disappearances were all significantly different between both periods except for AR4 (Table S11). For species numbers per altitudinal rank see Table S3.*

Table S10 *Relative colonisation and disappearance per altitudinal rank (AR) in the periods 1994-2004 and 2004-2014 on Mount Schrankogel.* ***a)*** *Mean and standard error (SE) of raw data and* ***b)*** *least-square mean (lsmean), standard error (SE) and 95% confidence interval (LCL and UCL), derived from generalized linear mixed effect models with function glmmadmb and a binomial distribution, of relative colonisation events per unoccupied plot and relative disappearance events per occupied plot for each species in different altitudinal ranks during two periods. For species numbers per altitudinal rank see Table S3.*

			(a) Raw data		(b) Model			
Period	Type	AR	mean	SE	lsmean	SE	LCL	UCL
1994-2004	Colonisation	1	0.14	0.01	0.17	2.23	0.03	0.82
2004-2014	Colonisation	1	0.07	0.01	0.08	2.16	0.02	0.37
1994-2004	Colonisation	2	0.07	0.00	0.03	2.84	0.00	0.25
2004-2014	Colonisation	2	0.07	0.00	0.04	2.81	0.01	0.31
1994-2004	Colonisation	3	0.04	0.00	0.01	2.93	0.00	0.06
2004-2014	Colonisation	3	0.03	0.00	0.01	2.90	0.00	0.05
1994-2004	Colonisation	4	0.00	0.00	0.00	4.13	0.00	0.01
2004-2014	Colonisation	4	0.00	0.00	0.00	3.49	0.00	0.01
1994-2004	Disappearance	1	-0.03	0.00	-0.02	1.64	-0.05	-0.01
2004-2014	Disappearance	1	-0.08	0.01	-0.06	1.55	-0.15	-0.03
1994-2004	Disappearance	2	-0.03	0.00	-0.04	2.00	-0.15	-0.01
2004-2014	Disappearance	2	-0.07	0.01	-0.10	1.78	-0.32	-0.03
1994-2004	Disappearance	3	-0.02	0.01	-0.02	2.07	-0.10	-0.01
2004-2014	Disappearance	3	-0.16	0.01	-0.17	1.70	-0.49	-0.06
1994-2004	Disappearance	4	-0.23	0.12	-0.22	3.31	-2.27	-0.02
2004-2014	Disappearance	4	-0.22	0.09	-0.19	2.16	-0.87	-0.04

Table S11 *Changes in relative colonisation and disappearance per altitudinal rank (AR) in the periods 1994-2004 and 2004-2014 on Mount Schrankogel. Mean changes (Odds ratio) in relative colonisation and disappearance events between the periods 1994 -2004 and 2004 -2014 with standard error (SE), z-ratio and p-value, derived from generalized linear mixed-effects models with the function glmmadmb and likelihood ratio tests. For species numbers per altitudinal rank see Table S3.*

AR	Type	Contrast	Odds ratio	SE	z-ratio	p-value
1	Colonisation	1994 – 2004 == 2004 – 2014	2.05	0.42	3.50	0.01
2	Colonisation	2004 – 2014 == 1994 – 2004	1.27	0.18	1.70	0.69
3	Colonisation	1994 – 2004 == 2004 – 2014	1.06	0.16	0.40	1.00
4	Colonisation	2004 – 2014 == 1994 – 2004	1.93	1.16	1.10	0.96
1	Disappearance	2004 – 2014 == 1994 – 2004	3.23	0.62	6.15	<.0001
2	Disappearance	2004 – 2014 == 1994 – 2004	2.65	0.84	3.08	0.04
3	Disappearance	2004 – 2014 == 1994 – 2004	6.90	2.73	4.88	<.0001
4	Disappearance	1994 – 2004 == 2004 – 2014	1.12	1.04	0.13	1.00

Vascular plant cover

Table S12 *Cover sum of vascular plant species in the survey years 1994, 2004 and 2014 on Mount Schrankogel.* ***a)*** *Mean and standard error (SE) of raw data and* ***b)*** *least-square mean (lsmean), standard error (SE), degrees of freedom (df) and 95% confidence interval (LCL and UCL) derived from linear mixed effects models, of the cover sum of the vascular plant species per plot in the three survey years. For plot numbers per block see Table S1.*

		(a) Raw data		(b) Model				
Year	Block	mean	SE	lsmean	SE	df	LCL	UCL
1994	All	24.33	0.95	25.28	2.76	13.90	19.36	31.19
2004	All	21.90	0.78	22.79	2.76	13.90	16.88	28.71
2014	All	20.83	0.74	21.78	2.76	13.90	15.86	27.70
1994	A	27.91	1.56	29.93	4.95	2.95	14.03	45.83
2004	A	25.11	1.32	27.00	4.95	2.95	11.10	42.90
2014	A	24.70	1.25	26.72	4.95	2.95	10.82	42.62
1994	B	21.27	1.43	24.19	5.71	3.03	6.10	42.28
2004	B	18.50	1.09	21.42	5.71	3.03	3.33	39.50
2014	B	16.55	1.04	19.46	5.71	3.03	1.38	37.55
1994	C	24.79	1.96	23.86	5.91	1.01	-48.61	96.33
2004	C	23.46	1.51	22.53	5.91	1.01	-49.94	95.01
2014	C	21.40	1.36	20.47	5.91	1.01	-52.01	92.94
1994	D	12.96	2.68	21.54	5.73	5.22	7.00	36.09
2004	D	13.15	2.44	21.73	5.73	5.22	7.19	36.28
2014	D	14.47	2.39	23.06	5.73	5.22	8.51	37.60

Table S13 *Changes in cover sum of vascular plant species in the periods 1994-2004, 1994-2014 and 2004-2014 on Mount Schrankogel. Effect sizes (estimate), 95% confidence interval (LCL and UCL) and p-values of the cover sum of the vascular plant species per plot between the initial survey 1994 and the resurveys 2004 and 2014, derived from linear mixed effects models. For plot numbers per block see Table S1.*

Block	Hypothesis	Estimate	LCL	UCL	p-value
All	2004 - 1994 == 0	-2.48	-2.91	-2.05	0.000
All	2014 - 1994 == 0	-3.50	-3.92	-3.07	0.000
All	2014 - 2004 == 0	-1.01	-1.44	-0.59	0.047
A	2004 - 1994 == 0	-2.94	-3.66	-2.21	0.000
A	2014 - 1994 == 0	-3.21	-3.93	-2.49	0.000
A	2014 - 2004 == 0	-0.27	-1.00	0.45	0.924
B	2004 - 1994 == 0	-2.77	-3.40	-2.14	0.000
B	2014 - 1994 == 0	-4.72	-5.35	-4.09	0.000
B	2014 - 2004 == 0	-1.95	-2.58	-1.32	0.006
C	2004 - 1994 == 0	-1.33	-2.21	-0.44	0.295
C	2014 - 1994 == 0	-3.39	-4.28	-2.51	0.001
C	2014 - 2004 == 0	-2.07	-2.95	-1.18	0.055
D	2004 - 1994 == 0	0.19	-1.31	1.68	0.991
D	2014 - 1994 == 0	1.51	0.02	3.01	0.574
D	2014 - 2004 == 0	1.32	-0.17	2.82	0.652

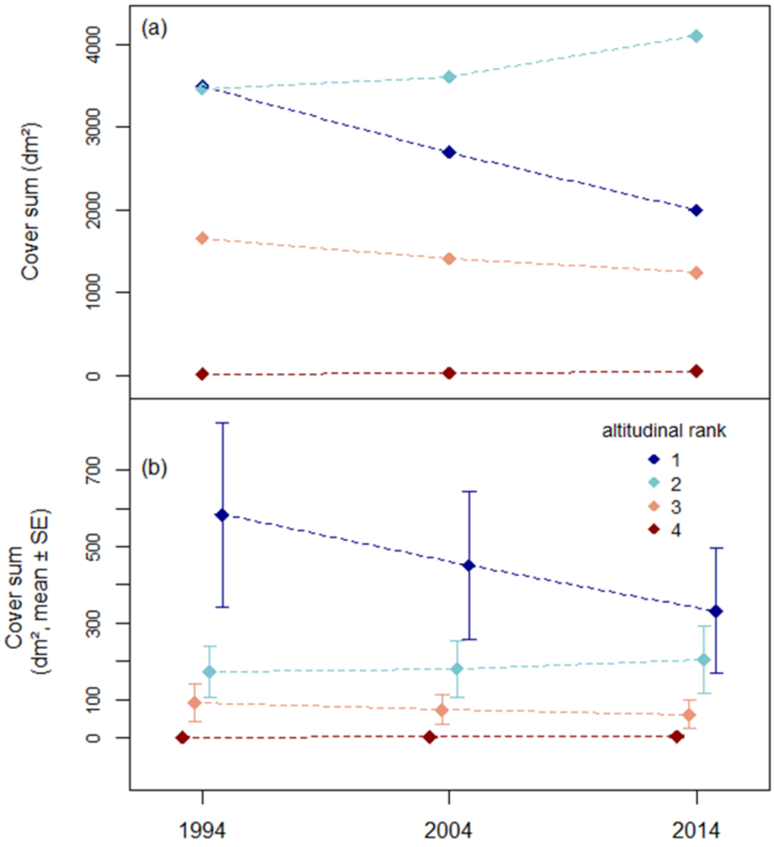

Fig. S5 *Cover sum of vascular plant species per altitudinal rank (AR) in the survey years 1994, 2004 and 2014 on Mount Schrankogel. a) Cover sum over all 355 plots for all species combined in each AR group, and b) mean and standard error of raw data of cover sum per species in each altitudinal rank (Table S14). In b) only the three years within altitudinal rank 1 were significantly different (linear mixed effects models; Table S15). For species numbers per altitudinal rank and year see Table S3.*

Table S14 *Cover sum of vascular plant species per altitudinal rank (AR) in the survey years 1994, 2004 and 2014 on Mount Schrankogel.* ***a)*** *Cover sum (dm²) over all plots, mean and standard error (SE) of raw data and* ***b)*** *least-square mean (lsmean), standard error (SE), degrees of freedom (df) and 95% confidence interval (LCL and UCL) derived from linear mixed effects models, of the cover sum per vascular plant species in the three survey years. For species numbers per altitudinal rank and year see Table S3.*

		(a) Raw data			(b) Model				
Year	AR	sum	mean	SE	lsmean	SE	df	LCL	UCL
1994	1	3496.73	582.79	240.35	582.79	109.56	60	363.66	801.92
2004	1	2704.51	450.75	194.68	450.75	109.56	60	231.62	669.88
2014	1	1995.14	332.52	163.36	332.52	109.56	60	113.39	551.65
1994	2	3465.12	173.26	67.70	173.26	60.01	60	53.23	293.28
2004	2	3611.52	180.58	73.72	180.58	60.01	60	60.55	300.60
2014	2	4107.83	205.39	87.19	205.39	60.01	60	85.37	325.41
1994	3	1659.93	92.22	50.82	82.35	58.95	62	-35.50	200.19
2004	3	1409.16	74.17	39.33	68.25	58.82	61	-49.35	185.85
2014	3	1243.54	62.18	35.64	58.91	58.68	61	-58.44	176.26
1994	4	13.76	2.29	1.14	-1.81	75.35	72	-152.00	148.38
2004	4	26.50	3.31	1.71	1.30	73.79	67	-145.98	148.59
2014	4	47.87	3.68	2.48	3.83	71.97	61	-140.08	147.74

Table S15 *Changes in mean cover sum of vascular plant species per altitudinal rank (AR) in the periods 1994-2004, 1994-2014 and 2004-2014 on Mount Schrankogel. Effect sizes (estimate), 95% confidence interval (LCL and UCL) and p-values of the mean cover sum (dm²) of the vascular plant species per AR between the initial survey 1994 and the resurveys 2004 and 2014, derived from linear mixed effects models. For species numbers per altitudinal rank and year see Table S3.*

AR	Hypothesis	Estimate	LCL	UCL	p-value
1	2004 - 1994 == 0	-132.04	-163.79	-100.28	0.004
1	2014 - 1994 == 0	-250.26	-282.02	-218.51	<.0001
1	2014 - 2004 == 0	-118.23	-149.99	-86.47	0.017
2	2004 - 1994 == 0	7.32	-10.07	24.71	1.000
2	2014 - 1994 == 0	32.14	14.74	49.53	0.787
2	2014 - 2004 == 0	24.82	7.42	42.21	0.955
3	2004 - 1994 == 0	-14.09	-32.42	4.23	1.000
3	2014 - 1994 == 0	-23.44	-41.75	-5.12	0.980
3	2014 - 2004 == 0	-9.34	-27.65	8.96	1.000
4	2004 - 1994 == 0	3.11	-29.87	36.09	1.000
4	2014 - 1994 == 0	5.64	-27.21	38.48	1.000
4	2014 - 2004 == 0	2.53	-24.86	29.91	1.000

Changes in community-based ecological indicators

Thermic indicator

Table S16 *Thermic indicator in the survey years 1994, 2004 and 2014 on Mount Schrankogel. Least-square mean (lsmean), standard error (SE), degrees of freedom (df, rounded to the nearest integer) and 95% confidence interval (LCL and UCL) of the thermic indicator per plot in the three survey years. As a proxy for the thermal preferences of a species the altitudinal rank (1 = subnival-nival, 2 = alpine-subnival, 3 = alpine, 4 = treeline-alpine species; Gottfried et al., 2012) was used. Data were analysed with linear mixed-effects models. For plot numbers per block see Table S1.*

Year	Block	lsmean	SE	df	LCL	UCL
1994	All	1.61	0.15	5	1.23	1.99
2004	All	1.67	0.15	5	1.29	2.06
2014	All	1.76	0.15	5	1.38	2.14
1994	A	1.61	0.19	4	1.08	2.14
2004	A	1.69	0.19	4	1.16	2.22
2014	A	1.80	0.19	4	1.27	2.33
1994	B	1.90	0.14	3	1.44	2.35
2004	B	1.94	0.14	3	1.48	2.39
2014	B	2.01	0.14	3	1.55	2.46
1994	C	1.79	0.27	1	-1.61	5.19
2004	C	1.85	0.27	1	-1.56	5.25
2014	C	1.92	0.27	1	-1.48	5.32
1994	D	1.19	0.06	21	1.05	1.32
2004	D	1.23	0.06	21	1.09	1.37
2014	D	1.25	0.06	21	1.12	1.39

Table S17 *Thermophilisation in the periods 1994-2004, 1994-2014 and 2004-2014 on Mount Schrankogel. Mean changes (estimate) in thermic indicator (Δ thermic indicator = thermophilisation) between 1994 and 2004, 1994 and 2014, and 2004 and 2014, with standard error (SE), 95% confidence interval (LCL and UCL) and p-value, derived from linear mixed-effects models and likelihood ratio tests. For plot numbers per block see Table S1.*

Block	Hypothesis	Estimate	SE	LCL	UCL	p-value
All	2004 - 1994 == 0	0.06	0.01	0.04	0.08	<0.001
All	2014 - 1994 == 0	0.15	0.01	0.13	0.17	<0.001
All	2014 - 2004 == 0	0.09	0.01	0.07	0.11	<0.001
A	2004 - 1994 == 0	0.08	0.01	0.05	0.11	<0.001
A	2014 - 1994 == 0	0.19	0.01	0.16	0.22	<0.001
A	2014 - 2004 == 0	0.11	0.01	0.08	0.14	<0.001
B	2004 - 1994 == 0	0.04	0.01	0.01	0.07	0.015
B	2014 - 1994 == 0	0.11	0.01	0.08	0.14	0.001
B	2014 - 2004 == 0	0.07	0.01	0.04	0.11	0.001
C	2004 - 1994 == 0	0.06	0.02	0.02	0.10	0.003
C	2014 - 1994 == 0	0.13	0.02	0.09	0.17	0.001
C	2014 - 2004 == 0	0.07	0.02	0.03	0.11	0.001
D	2004 - 1994 == 0	0.04	0.02	-0.01	0.10	0.138
D	2014 - 1994 == 0	0.06	0.02	0.01	0.12	0.015
D	2014 - 2004 == 0	0.02	0.02	-0.03	0.07	0.651

Table S18 *Changes in thermophilisation between the periods 1994-2004 and 2004-2014 on Mount Schrankogel. Effect sizes (estimate) of changes in thermophilisation (Δ thermic indicator) from the first (1994-2004) to the second (2004-2014) decade of survey, with 95% confidence interval (LCL and UCL) and p-value, derived from linear mixed-effects models and likelihood ratio test. For plot numbers per block see Table S1.*

Block	Estimate	LCL	UCL	p-value
All	0.03	0.01	0.05	0.009
A	0.03	-0.01	0.06	0.108
B	0.04	0.01	0.07	0.020
C	0.02	-0.02	0.06	0.429
D	-0.02	-0.07	0.03	0.344

Soil moisture indicator

Table S19 *Soil moisture indicator in the survey years 1994, 2004 and 2014 on Mount Schrankogel. Least-square mean (lsmean), standard error (SE), rounded degrees of freedom (df) and 95% confidence interval (LCL and UCL) of the soil moisture indicator per plot in the three survey years. As a proxy for the soil moisture preferences of a species the indicator value for soil moisture (1 = very dry to 4 = very moist; Landolt et al., 2010) was used. Data were analysed with linear mixed-effects models. For plot numbers per block see Table S1.*

Year	Block	lsmean	SE	df	LCL	UCL
1994	All	2.95	0.13	5	2.62	3.27
2004	All	2.89	0.13	5	2.57	3.22
2014	All	2.79	0.13	5	2.46	3.12
1994	A	2.97	0.10	4	2.68	3.26
2004	A	2.92	0.10	4	2.63	3.21
2014	A	2.77	0.10	4	2.49	3.06
1994	B	2.79	0.14	3	2.35	3.24
2004	B	2.75	0.14	3	2.31	3.20
2014	B	2.68	0.14	3	2.24	3.12
1994	C	2.68	0.14	1	1.01	4.36
2004	C	2.60	0.14	1	0.92	4.27
2014	C	2.53	0.14	1	0.86	4.20
1994	D	3.32	0.06	23	3.20	3.44
2004	D	3.27	0.06	23	3.15	3.39
2014	D	3.20	0.06	23	3.08	3.32

Table S20 *Change in soil moisture indicator in the periods 1994-2004, 1994-2014 and 2004-2014 on Mount Schrankogel. Least-square mean changes (estimate) in soil moisture indicator between 1994 and 2004, 1994 and 2014, and 2004 and 2014, with standard error (SE), 95% confidence interval (LCL and UCL) and p-value, derived from linear mixed-effects models and likelihood ratio test. For plot numbers per block see Table S1.*

Block	Hypothesis	Estimate	SE	LCL	UCL	p-value
All	2004 - 1994 == 0	-0.05	0.01	-0.07	-0.03	<0.001
All	2014 - 1994 == 0	-0.16	0.01	-0.18	-0.14	<0.001
All	2014 - 2004 == 0	-0.11	0.01	-0.13	-0.08	<0.001
A	2004 - 1994 == 0	-0.05	0.02	-0.09	-0.01	0.003
A	2014 - 1994 == 0	-0.20	0.02	-0.24	-0.16	0.001
A	2014 - 2004 == 0	-0.15	0.02	-0.19	-0.11	0.001
B	2004 - 1994 == 0	-0.04	0.01	-0.07	-0.01	0.001
B	2014 - 1994 == 0	-0.11	0.01	-0.14	-0.08	<0.001
B	2014 - 2004 == 0	-0.07	0.01	-0.10	-0.04	<0.001
C	2004 - 1994 == 0	-0.08	0.02	-0.13	-0.03	0.001
C	2014 - 1994 == 0	-0.15	0.02	-0.20	-0.10	0.001
C	2014 - 2004 == 0	-0.07	0.02	-0.12	-0.02	0.004
D	2004 - 1994 == 0	-0.05	0.03	-0.13	0.02	0.206
D	2014 - 1994 == 0	-0.12	0.03	-0.19	-0.04	<0.001
D	2014 - 2004 == 0	-0.07	0.03	-0.14	0.01	0.098

Table S21 *Changes in Δ soil moisture indicator between the periods 1994-2004 and 2004-2014 on Mount Schrankogel. Effect sizes (estimate) of Δ soil moisture indicator from the first (1994-2004) to the second (2004-2014) decade of survey, with 95% confidence intervals (LCL and UCL) and p-value, derived from linear mixed-effects models and likelihood ratio test. For plot numbers per block see Table S1.*

Block	Estimate	LCL	UCL	p-value
All	-0.05	-0.07	-0.03	<0.001
A	-0.10	-0.13	-0.06	<0.001
B	-0.03	-0.05	-0.01	0.016
C	0.01	-0.03	0.06	0.543
D	-0.01	-0.06	0.04	0.638

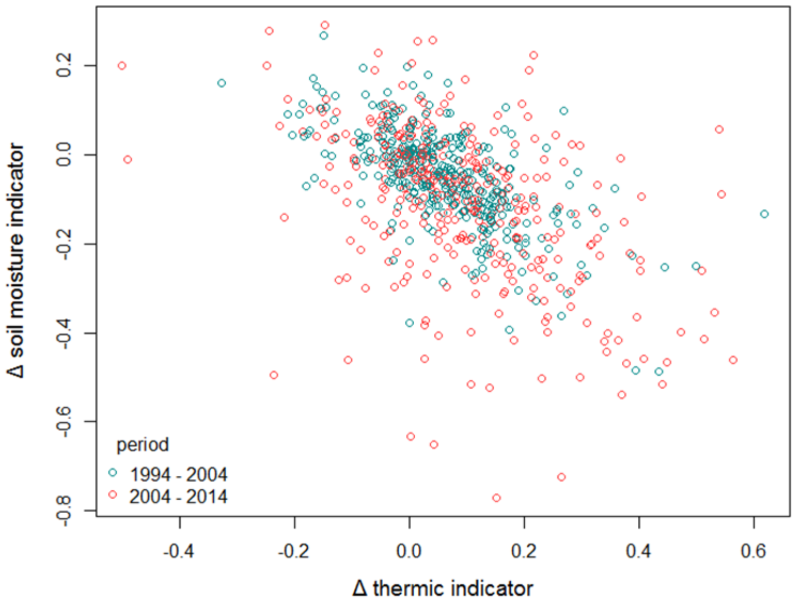

Fig. S6 *Correlation between the changes of thermic indicator and changes of soil moisture indicator per plot in the periods 1994-2004 and 2004-2014 on Mount Schrankogel. The thermic indicator per plot was calculated as the mean altitudinal rank (1 = subnival-nival, 2 = alpine-subnival, 3 = alpine, 4 = treeline-alpine species) weighted with the respective species' cover (Gottfried et al., 2012). The soil moisture indicator per plot was calculated as the mean of indicator values for soil moisture (1 = very dry to 4 = very moist; Landolt et al., 2010) weighted with the respective species' cover. The correlation was highly significant (linear mixed effect model; p <0.0001).*

Topography and elevation

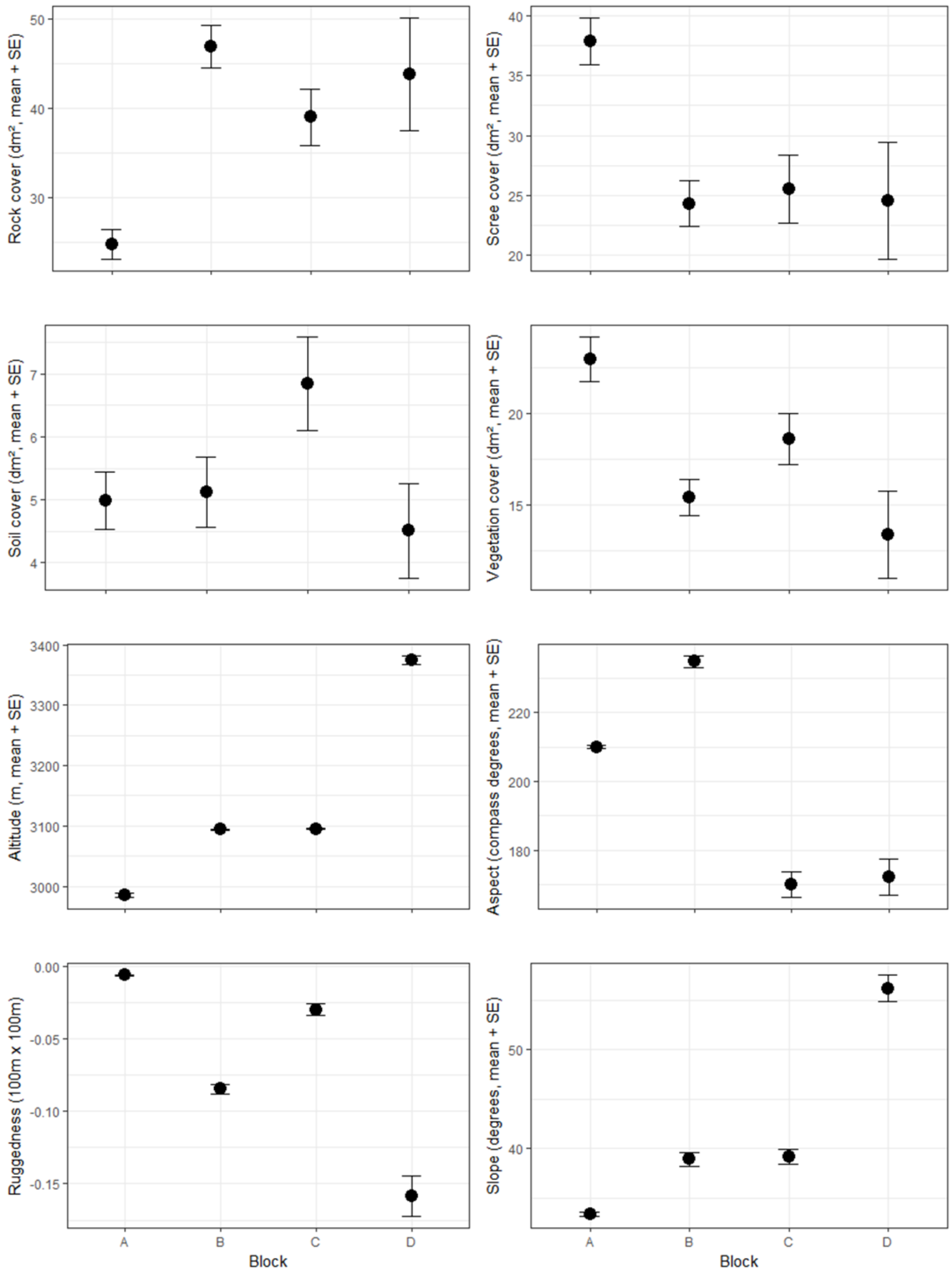

Fig. S7 *Topographic parameters per block on Mount Schrankogel. Means of estimated top cover of surface types per plot (rock, scree, bare soil and vegetation) and topographic parameters (altitude, aspect, slope and ruggedness) derived from a digital elevation model (© Land Tirol). Ruggedness was calculated as standard deviation of elevation with a 100 m x 100 m raster per plot by using ArcGIS 10.3. for Desktop, Esri Inc. For plot numbers per block see Table S1.*

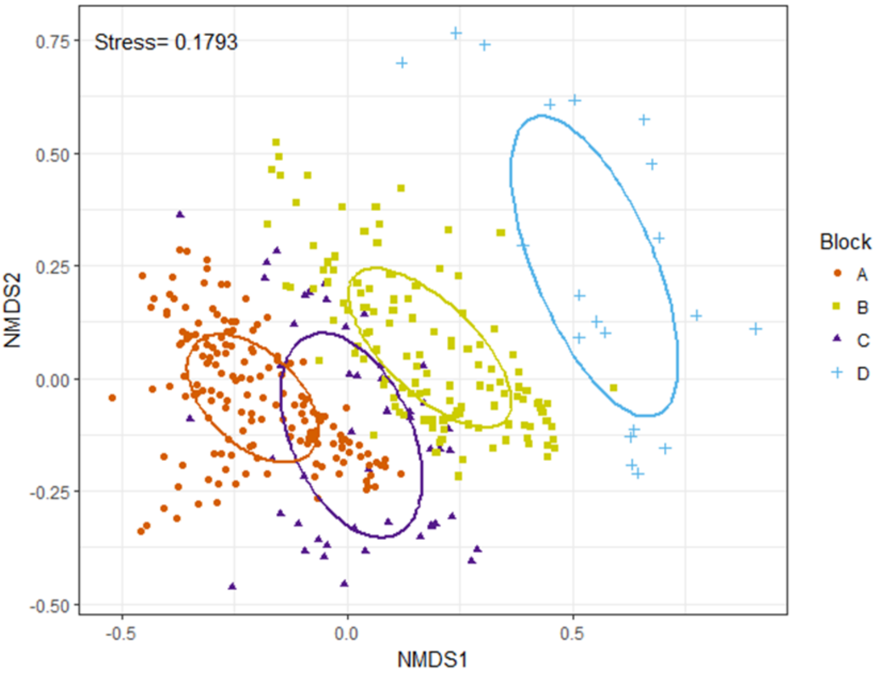

Fig. S8 *Non-metric multidimensional scaling (NMDS) of permanent plots on Mount Schrankogel, which was fitted using rescaled topographic parameters (altitude, aspect, slope, ruggedness) derived from a digital elevation model (© Land Tirol) and estimated cover of main surface types per plot (rock, scree, bare soil and vegetation). Ellipses represent the standard deviations of each plot score within each block. For plot numbers per block see Table S1.*

Table S22 *Differences among blocks on Mount Schrankogel. Differences (estimate*) among *blocks (contrast), standard error (SE), degrees of freedom (df) and p-values derived from linear mixed effect models with the first axis of a non-metric multidimensional scaling of topographic parameters (Fig. S8) as response, block as fixed effect and transect as random effect.* For plot *numbers per block see Table S1.*

Contrast	Estimate	SE	df	p.value
A – B	0.44	0.08	8.87	0.001
A – C	0.25	0.09	8.46	0.099
A – D	0.81	0.08	13.50	<0.001
B – C	0.19	0.10	8.56	0.274
B – D	0.37	0.08	13.10	0.003
C – D	0.56	0.10	11.01	<0.001

Dataset 1994 – 2014 (n_{plot} = 654)

Vascular plant species richness

Table S23 *Vascular plant species richness in the survey years 1994 and 2014 on Mount Schrankogel.* ***a)*** *Mean and standard error (SE) of raw data and* ***b)*** *least-square mean (lsmean), standard error (SE), degrees of freedom (df) and 95% confidence interval (LCL and UCL), derived from generalized linear mixed effect models with a penalized quasi-likelihood estimation and a Poisson distribution, of the species richness per plot in the two survey years. For plot numbers per block see Table S1.*

		(a) Raw data		(b) Model				
Year	Block	mean	SE	lsmean	SE	df	LCL	UCL
1994	All	9.95	0.17	8.02	1.08	3	5.22	12.32
2014	All	12.64	0.19	10.19	1.37	3	6.64	15.65
1994	A	10.58	0.22	9.47	1.63	4	5.88	15.25
2014	A	14.18	0.24	12.69	2.18	4	7.88	20.43
1994	B	8.36	0.27	7.83	1.12	3	4.97	12.33
2014	B	9.75	0.28	9.13	1.30	3	5.80	14.37
1994	C	12.27	0.43	11.82	0.44	1	7.33	19.07
2014	C	14.80	0.47	14.26	0.53	1	8.92	22.81
1994	D	5.69	0.43	5.21	0.58	25	4.14	6.54
2014	D	6.54	0.49	5.98	0.66	25	4.77	7.50

Table S24 *Changes in vascular plant species richness in the period 1994-2014 on Mount Schrankogel. Effect sizes (estimate), 95% confidence interval (LCL and UCL) and p-values of the species richness per plot between the initial survey 1994 and the resurvey 2014, derived from generalized linear mixed effect models with a penalized quasi-likelihood estimation and a Poisson distribution. For plot numbers per block see Table S1.*

Block	Estimate	LCL	UCL	p-value
All	0.24	0.22	0.26	<0.001
A	0.29	0.26	0.32	<0.001
B	0.15	0.12	0.19	<0.001
C	0.19	0.14	0.23	<0.001
D	0.14	0.04	0.24	0.011

Colonisations and disappearances

Table S25 *Number of colonising and disappearing vascular plant species in the period 1994-2014 on Mount Schrankogel.* ***a)*** *Mean and standard error (SE) of raw data and* ***b)*** *least-square mean (lsmean), standard error (SE) and asymptotic 95% confidence interval (LCL and UCL), derived from negative binomial generalized linear mixed-effects models, of numbers of colonising and disappearing species per plot between the initial survey 1994 and the resurvey 2014. For plot numbers per block see Table S1.*

		(a) Raw data		(b) Model			
Type	Block	mean	SE	lsmean	SE	LCL	UCL
Colonisations	All	3.57	0.10	2.54	0.58	1.624	3.984
Disappearances	All	0.88	0.04	0.63	0.15	0.401	0.995
Colonisations	A	4.51	0.14	4.39	0.34	3.765	5.121
Disappearances	A	0.91	0.06	0.89	0.08	0.743	1.070
Colonisations	B	2.20	0.12	2.08	0.32	1.532	2.817
Disappearances	B	0.82	0.08	0.77	0.13	0.553	1.067
Colonisations	C	3.53	0.24	3.23	0.56	2.296	4.550
Disappearances	C	1.00	0.13	0.92	0.18	0.623	1.350
Colonisations	D	1.31	0.22	1.30	0.25	0.890	1.902
Disappearances	D	0.46	0.14	0.46	0.14	0.255	0.826

Table S26 *Differences between numbers of colonising and disappearing vascular plant species in the period 1994-2014 on Mount Schrankogel. Effect sizes (estimate), 95% confidence interval (LCL and UCL) and p-values of differences of numbers of colonising and disappearing species per plot over the entire study area and for each block between 1994 and 2014, derived from negative binomial generalized linear mixed-effects models with an ANOVA likelihood ratio test. For plot numbers per block see Table S1.*

Block	Estimate	LCL	UCL	p-value
All	1.39	1.28	1.51	<0.001
A	1.59	1.45	1.74	<0.001
B	1.00	0.77	1.22	<0.001
C	1.26	0.97	1.55	<0.001
D	1.04	0.32	1.76	0.001

Changes in community-based ecological indicators

Thermic indicator

Table S27 *Thermic indicator in the survey years 1994 and 2014 on Mount Schrankogel. Least-square mean (lsmean), standard error (SE), degrees of freedom (df, rounded to the nearest integer) and 95% confidence interval (LCL and UCL) of the thermic indicator per plot of the years 1994 and 2014. As a proxy for the thermal preferences of a species the altitudinal rank (1 = subnival-nival, 2 = alpine-subnival, 3 = alpine, 4 = treeline-alpine species; Gottfried et al., 2012) was used. Data were analysed with linear mixed-effects models. For plot numbers per block see Table S1.*

Year	Block	lsmean	SE	df	LCL	UCL
1994	All	1.56	0.13	4	1.21	1.90
2014	All	1.71	0.13	4	1.37	2.05
1994	A	1.60	0.18	4	1.11	2.09
2014	A	1.77	0.18	4	1.27	2.26
1994	B	1.80	0.08	3	1.53	2.07
2014	B	1.93	0.08	3	1.66	2.20
1994	C	1.60	0.09	1	0.51	2.70
2014	C	1.79	0.09	1	0.70	2.89
1994	D	1.29	0.13	6	0.98	1.60
2014	D	1.36	0.13	6	1.05	1.67

Table S28 *Thermophilisation in the period 1994-2014 on Mount Schrankogel. Mean changes (estimate) in thermic indicator (Δ indicator = thermophilisation) between 1994 and 2014, with 95% confidence interval (LCL and UCL) and p-value, derived from linear mixed-effects models and likelihood ratio tests. For plot numbers per block see Table S1.*

Block	Estimate	LCL	UCL	p-value
All	0.15	0.14	0.17	<0.001
A	0.17	0.14	0.19	<0.001
B	0.12	0.09	0.16	<0.001
C	0.19	0.15	0.23	<0.001
D	0.07	0.02	0.12	0.009

Soil moisture indicator

Table S29 *Soil moisture indicator in the survey years 1994 and 2014 on Mount Schrankogel. Least-square mean (lsmean), standard error (SE), rounded degrees of freedom (df) and 95% confidence interval (LCL and UCL) of the soil moisture indicator per plot in the three survey years. As a proxy for the soil moisture preferences of a species the indicator value for soil moisture (1 = very dry to 4 = very moist; Landolt et al., 2010) was used. Data were analysed with linear mixed-effects models. For plot numbers per block see Table S1.*

Year	Block	lsmean	SE	df	LCL	UCL
1994	All	2.97	0.10	4	2.68	3.26
2014	All	2.83	0.10	4	2.55	3.12
1994	A	2.97	0.10	4	2.69	3.24
2014	A	2.83	0.10	4	2.55	3.10
1994	B	2.82	0.09	3	2.53	3.12
2014	B	2.71	0.09	3	2.41	3.01
1994	C	2.82	0.04	1	1.94	3.69
2014	C	2.64	0.04	1	1.76	3.51
1994	D	3.22	0.14	6	2.88	3.55
2014	D	3.10	0.14	6	2.76	3.43

Table S30 *Changes in soil moisture in the period 1994-2014 on Mount Schrankogel. Mean changes (estimate) in soil moisture indicator between 1994 and 2014, with 95% confidence interval (LCL and UCL) and p-value, derived from linear mixed-effects models and likelihood ratio test. For plot numbers per block see Table S1.*

Block	Estimate	LCL	UCL	p-value
All	-0.14	-0.16	-0.12	<0.001
A	-0.14	-0.17	-0.11	<0.001
B	-0.12	-0.14	-0.09	<0.001
C	-0.18	-0.23	-0.14	<0.001
D	-0.12	-0.19	-0.05	0.002

References

Burnham KP, Anderson DR. 2002. *Model selection and multimodel inference: a practical information-theoretic approach*. New York: Springer-Verlag.

Gottfried M, Pauli H, Futschik A, Akhalkatsi M, Barancok P, Benito Alonso JL, Coldea G, Dick J, Erschbamer B, Fernandez Calzado MR, et al. 2012. Continent-wide response of mountain vegetation to climate change. *Nature Climate* Change 2: 111-115.

Landolt E, Bäumler B, Erhardt A, Hegg O, Klötzli F, Lämmler W, Nobis M, Rudmann-Maurer K, Schweingruber FH, Theurillat J-P, et al. 2010. *Flora indicativa: Ökologische Zeigerwerte und biologische Kennzeichen zur Flora der Schweiz und der Alpen / Ecological indicator values and biological attributes of the Flora of Switzerland and the Alps*. Bern: Haupt Verlag.
